# Metabolic Vulnerability and Regenerative Failure of the External Urethral Sphincter: A Ferroptosis-Associated Framework for Stress Urinary Incontinence

**DOI:** 10.3390/biom16091376

**Published:** 2026-09-21

**Authors:** Shuo Sun, Jialiang Liu, Xiaoying Pan, Yong Yang, Zhitao Wei

**Affiliations:** 1 Changchun University of Chinese Medicine, Changchun 130117, China; 24111709157@stu.ccucm.edu.cn (S.S.); liujialiang.2006@163.com (J.L.); 23111570913@stu.ccucm.edu.cn (X.P.); 2 The Affiliated Hospital of Changchun University of Chinese Medicine, Changchun 130021, China

**Keywords:** external urethral sphincter, metabolic vulnerability, tissue resilience, ferroptosis, regenerative failure, stress urinary incontinence

## Abstract

Stress urinary incontinence (SUI) has long been understood as a structural failure of pelvic floor support. While this view explains acute anatomical damage, it offers little insight into why functionally similar injuries produce markedly different long-term outcomes, or why sphincteric deterioration often progresses years after the initial insult. In this review, we examine the possibility that progressive external urethral sphincter (EUS) degeneration reflects an underlying metabolic vulnerability. The EUS is a striated muscle of unusual metabolic demand, sustaining continuous basal tone through oxidative phosphorylation while retaining rapid contractile capacity. Its membranes are enriched in polyunsaturated fatty acids and subjected to recurrent ischemia–reperfusion, features that are hypothesized to limit redox buffering capacity. We discuss how parturition trauma, aging, obesity, and neurogenic injury may converge to disrupt iron homeostasis, impair the GSH/GPX4 antioxidant axis, and exhaust satellite cell-mediated repair. Crucially, direct evidence for ferroptosis in the EUS remains limited. We therefore integrate EUS-specific findings with evidence from skeletal muscle and other tissues to propose a hypothesis-generating framework. Rather than treating ferroptosis-associated stress as an isolated cell death pathway, we consider it a candidate biochemical nexus linking metabolic stress to fibro-adipogenic niche remodeling. By shifting focus from mechanical injury to eroding tissue resilience, this framework generates testable hypotheses regarding biomarker development and potential therapies aimed at restoring the adaptive capacity of the EUS.

## 1. Introduction

Stress urinary incontinence (SUI) is one of the most prevalent pelvic floor disorders in adult women, affecting an estimated 25–45% of women, with prevalence increasing substantially with age and after menopause [1,2,3]. SUI substantially compromises quality of life, restricts physical and social activity, and imposes a considerable healthcare burden. Despite its high prevalence, the biological processes that determine whether pelvic floor injury resolves, persists, or progresses remain incompletely understood. Current conservative and surgical treatments can effectively improve continence in many women, but they primarily compensate for or restore sphincteric function rather than reverse the underlying biological processes that may drive progressive tissue degeneration.

The prevailing view of SUI pathogenesis has long been anchored in biomechanics. Pregnancy, vaginal delivery, chronic pelvic loading, and age-related connective tissue changes can induce mechanical injury, denervation, vascular disruption, and extracellular matrix damage [4,5,6]. This structural framework effectively explains acute anatomical injury and has shaped contemporary therapeutic strategies. However, it leaves an important clinical observation incompletely explained: women exposed to apparently comparable injuries may experience markedly different long-term trajectories, and functional deterioration may become clinically evident years or decades after the initial insult [7,8]. This temporal and inter-individual disconnect constitutes a persistent degeneration paradox, suggesting that the long-term fate of the external urethral sphincter (EUS) cannot be determined solely by the magnitude of the initiating mechanical insult.

Recent advances in muscle biology suggest that tissue outcome depends not only on the severity of acute injury but also on intrinsic metabolic resilience and regenerative capacity. The EUS is a specialized striated muscle that must maintain continuous basal tone while retaining the capacity for rapid, forceful contraction. This physiological duality imposes substantial energetic demands and depends on sustained mitochondrial oxidative metabolism and ATP production [9,10,11]. Consistent with this metabolic specialization, metabolomic studies of human EUS myoblasts indicate that mitochondrial pyruvate transport and tricarboxylic acid cycle activity are essential for myogenic differentiation [12]. Such persistent oxidative activity may also increase exposure to reactive oxygen species and place greater demands on endogenous redox-buffering systems. Together, these features raise the possibility that the EUS represents a metabolically constrained regenerative niche in which relatively modest additional metabolic stress may compromise tissue repair.

This concept provides a potential link to ferroptosis-associated metabolic stress. Ferroptosis is an iron-dependent form of regulated cell death driven by phospholipid peroxidation and normally restrained by antioxidant systems including the System Xc^−^–glutathione–GPX4 axis [13,14,15]. The EUS may possess several characteristics that could increase its susceptibility to lipid peroxidative injury, including sustained oxidative metabolism, enrichment of polyunsaturated membrane lipids, and recurrent ischemia–reperfusion associated with pelvic floor loading [16,17,18]. Importantly, however, direct evidence establishing ferroptosis as a causal mechanism of EUS degeneration in SUI remains limited. We therefore do not propose ferroptosis as an established cause of SUI. Rather, ferroptosis-associated metabolic stress is considered here as a candidate biochemical convergence mechanism within a broader network involving oxidative stress, inflammation, cellular senescence, impaired myogenic regeneration, and fibro-adipogenic remodeling.

In this review, we advance a metabolic vulnerability framework in which progressive EUS dysfunction reflects an imbalance between cumulative metabolic stress and declining tissue resilience. We examine how parturition-related injury, aging, obesity, and neurogenic or vascular insults may progressively reduce the capacity of the EUS to maintain redox homeostasis and regenerate after injury, and how ferroptosis-associated signaling may contribute to the transition from acute damage to persistent regenerative failure. To distinguish established findings from mechanistic inference, supporting evidence is classified throughout the review as direct EUS/SUI evidence (D), muscle-associated evidence (M), cross-tissue evidence (X), or conceptual inference (H). This distinction is particularly important because much of the iron–lipid–redox machinery discussed in this framework is currently supported by M/X/H evidence rather than direct EUS validation. Accordingly, this article is intended as a hypothesis-generating conceptual review rather than a systematic synthesis of EUS-specific experimental evidence, with the goal of generating testable hypotheses for biomarker discovery and therapeutic strategies aimed at restoring EUS regenerative resilience. Mechanisms supported by direct EUS evidence are explicitly distinguished from those inferred from skeletal muscle or non-muscle systems, and the proposed framework is presented as a testable hypothesis rather than an established causal model.

## 2. Beyond Mechanical Injury: Redefining SUI as a Disorder of Regenerative Resilience

Dysfunction of the external urethral sphincter (EUS) represents a major pathological component of stress urinary incontinence (SUI) and age-related decline of lower urinary tract function. Despite extensive characterization of the anatomical deficits underlying these conditions, they remain highly prevalent among adult women and elderly populations, profoundly compromising patients’ quality of life and imposing substantial socio-economic burdens [1,2,3]. Contemporary interventions, ranging from pelvic floor physical therapy to surgical sling reconstructions, provide necessary mechanical support and alleviate symptoms; however, their long-term efficacy may be limited by progressive loss of regenerative capacity and biological deterioration of the EUS tissue itself.

Historically, the onset and progression of pelvic floor dysfunction have been interpreted almost exclusively through the lens of structural trauma. Risk factors such as pregnancy, vaginal childbirth, and chronic pelvic loading have been conceptualized as initiating mechanical injury, denervation, vascular disruption, and extracellular matrix (ECM) tearing [4,5,6]. While these classical mechanisms elegantly account for the acute phase of anatomical damage, they leave an important conceptual gap: they fail to explain the temporal dissociation between the initial injury and the onset of clinical symptoms.

This discrepancy gives rise to the persistent degeneration paradox. If mechanical injury alone were the sole determinant of EUS dysfunction, the trajectory of the disease should closely mirror the timeline of the trauma. Yet, clinically, while the most severe mechanical insults occur during parturition, the peak incidence of SUI often emerges years after the initial insult, particularly during aging-associated hormonal and tissue remodeling transitions [7]. Furthermore, individuals subjected to comparable degrees of obstetric trauma exhibit markedly divergent clinical trajectories—some achieve complete physiological restoration, whereas others undergo progressive muscle fiber loss, diminished regenerative capacity, and continuous functional deterioration long after the initial structural lesions have theoretically stabilized [8]. This suggests a fundamental tenet that must reshape our pathophysiological models: injury initiates degeneration, but regenerative capacity determines the disease trajectory. The long-term fate of the EUS is not dictated merely by the magnitude of the mechanical insult, but by progressive loss of intrinsic tissue homeostasis and the inability of the tissue to effectively resolve injury.

To resolve the persistent degeneration paradox, it is necessary to pivot from a purely biomechanical view of pelvic floor trauma to a biological framework centered on tissue resilience. Tissue resilience in the context of striated muscle refers to the intrinsic adaptive capacity of the tissue to withstand stress, buffer metabolic perturbations, and mount a successful regenerative response following injury.

When the EUS is subjected to repeated or cumulative stressors, the severity of the clinical outcome is mediated by the integrity of these reserve systems. A resilient microenvironment can efficiently clear damaged cells, neutralize oxidative stress, and activate satellite cells to restore the functional myofiber network. However, when metabolic resilience is compromised—whether by aging, systemic metabolic disease, or recurrent local hypoxia—the tissue loses its adaptive flexibility. Consequently, the injury response becomes maladaptive. Instead of undergoing functional repair, the EUS enters a state of chronic, unresolved stress, potentially driving the transition from adaptive regeneration to maladaptive, fibro-adipogenic degeneration. Recognizing this shift requires us to reconceptualize SUI not as an unhealed mechanical wound, but as an active, progressive failure of the local regenerative niche.

## 3. The External Urethral Sphincter as a Metabolically Vulnerable Regenerative Niche

For decades, investigations into EUS dysfunction have centered predominantly on structural alterations following acute injury. However, the trajectory of tissue recovery or degeneration is shaped not solely by the magnitude of extrinsic insults, but by the intrinsic biological properties of the EUS as an integrated regenerative niche. This functional niche comprises mature myofibers, resident satellite cells, vascular endothelial cells, immune populations, and supporting stromal cells, whose collective metabolic and redox properties shape the tissue’s baseline resilience to stress. To understand why regenerative failure occurs so prominently in the EUS, one must examine its unique biological microenvironment. The EUS represents a metabolically demanding regenerative niche. The biological features below represent intrinsic properties of the EUS that are either directly documented (D) or inferred from comparative muscle physiology (M). Where ferroptosis susceptibility is discussed, we explicitly note whether lipidomic or redox characteristics have been directly profiled in EUS tissue.

### 3.1. High Energetic Demand

**Figure 1:**
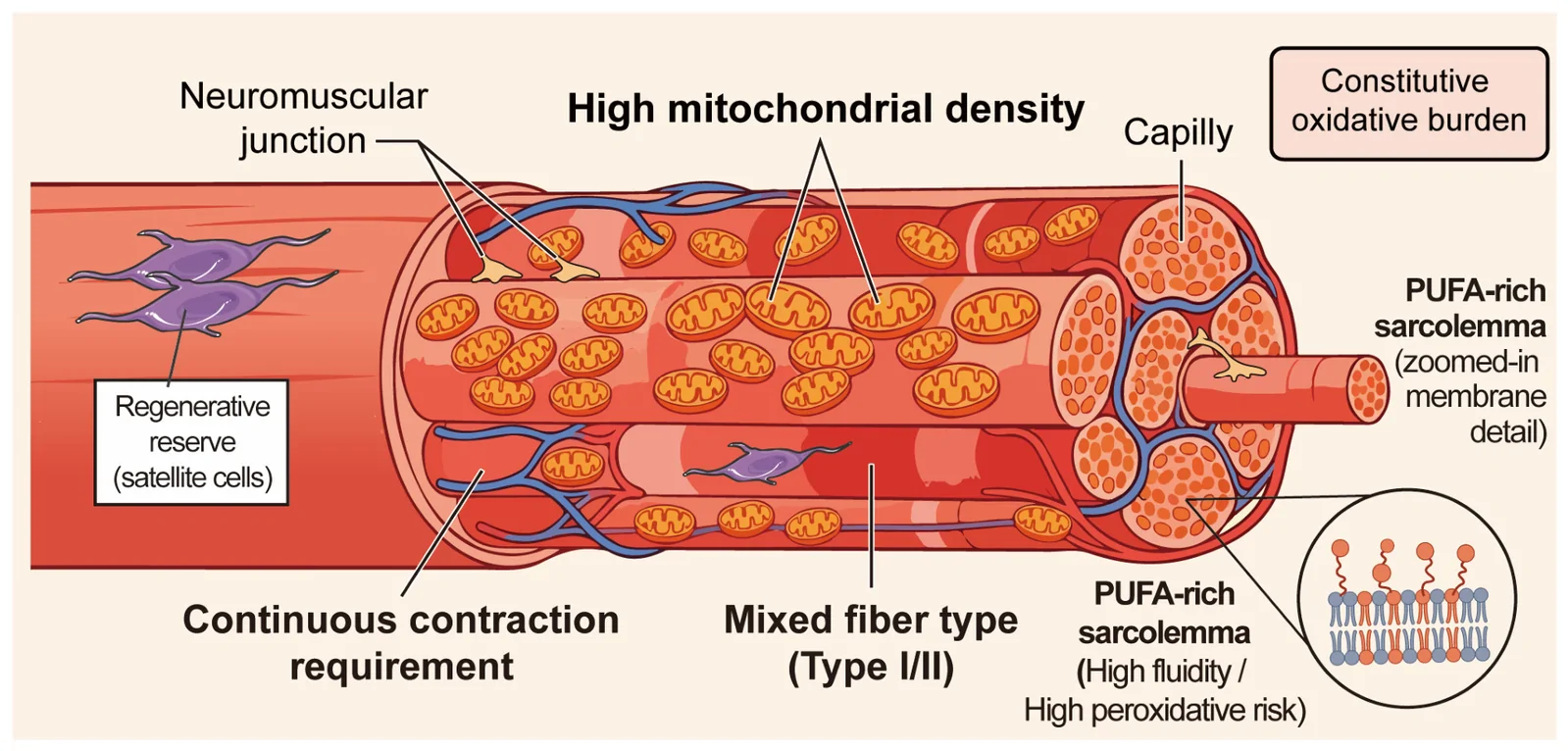
Physiological architecture and metabolic characteristics of the EUS microenvironment. Features such as high mitochondrial density, mixed fiber type composition, and PUFA-rich sarcolemma are empirically documented in EUS or comparative striated muscle (D/M). The ‘constitutive oxidative burden’ and ‘high peroxidative risk’ labels represent intrinsic biological properties; their direct quantification in native human EUS tissue remains incomplete.

The dual-fiber composition of the EUS is evolutionarily tuned to support its dual physiological role: slow-twitch fibers maintain chronic basal closure tone, while fast-twitch fibers mediate rapid, forceful contractions in response to abrupt intra-abdominal pressure elevations [9,10]. Unlike the appendicular skeletal muscles, which alternate between periods of intense exertion and prolonged rest, the EUS requires uninterrupted metabolic input throughout the lifespan, resulting in persistently high energy demand largely supported by mitochondrial oxidative phosphorylation (OXPHOS) (Figure 1) [9,11].

Sustained mitochondrial respiration inevitably produces reactive oxygen species (ROS) as a byproduct of electron transport chain activity, primarily superoxide anions and hydrogen peroxide (D for EUS mitochondrial density and continuous contraction requirement; H for quantitative ROS flux in native human EUS tissue). Under physiological conditions, a multi-layered antioxidant network efficiently scavenges excess ROS to preserve redox homeostasis. This equilibrium is, however, inherently fragile. Emerging human EUS metabolic data provide direct support for this metabolic dependence. A metabolomic study of human EUS myoblasts (US2KD cell line) demonstrated that mitochondrial pyruvate transport and tricarboxylic acid (TCA) cycle activity are non-negotiable prerequisites for myogenic differentiation [12]. Notably, while high-level oxidative metabolism is essential for normal EUS function, it also predisposes the tissue to cumulative oxidative damage. Furthermore, metabolic heterogeneity across fiber types may further shape differential vulnerability within the niche [19,20]. Oxidative fibers possess more extensive mitochondrial networks and higher oxygen flux, resulting in fundamentally distinct ROS generation patterns and stress response profiles compared to glycolytic fibers [11,21].

### 3.2. Limited Regenerative Reserve

The EUS possesses limited intrinsic adaptive capacity to buffer the burden of continuous physiological and mechanical demands. While resident satellite cells are required for structural renewal, their functional integrity progressively declines with aging, metabolic dysfunction, and chronic stress exposure. Consequently, these exact biological characteristics fundamentally constrain the metabolic flexibility required to maintain cellular homeostasis.

### 3.3. Redox-Sensitive Metabolic Architecture

**Figure 2:**
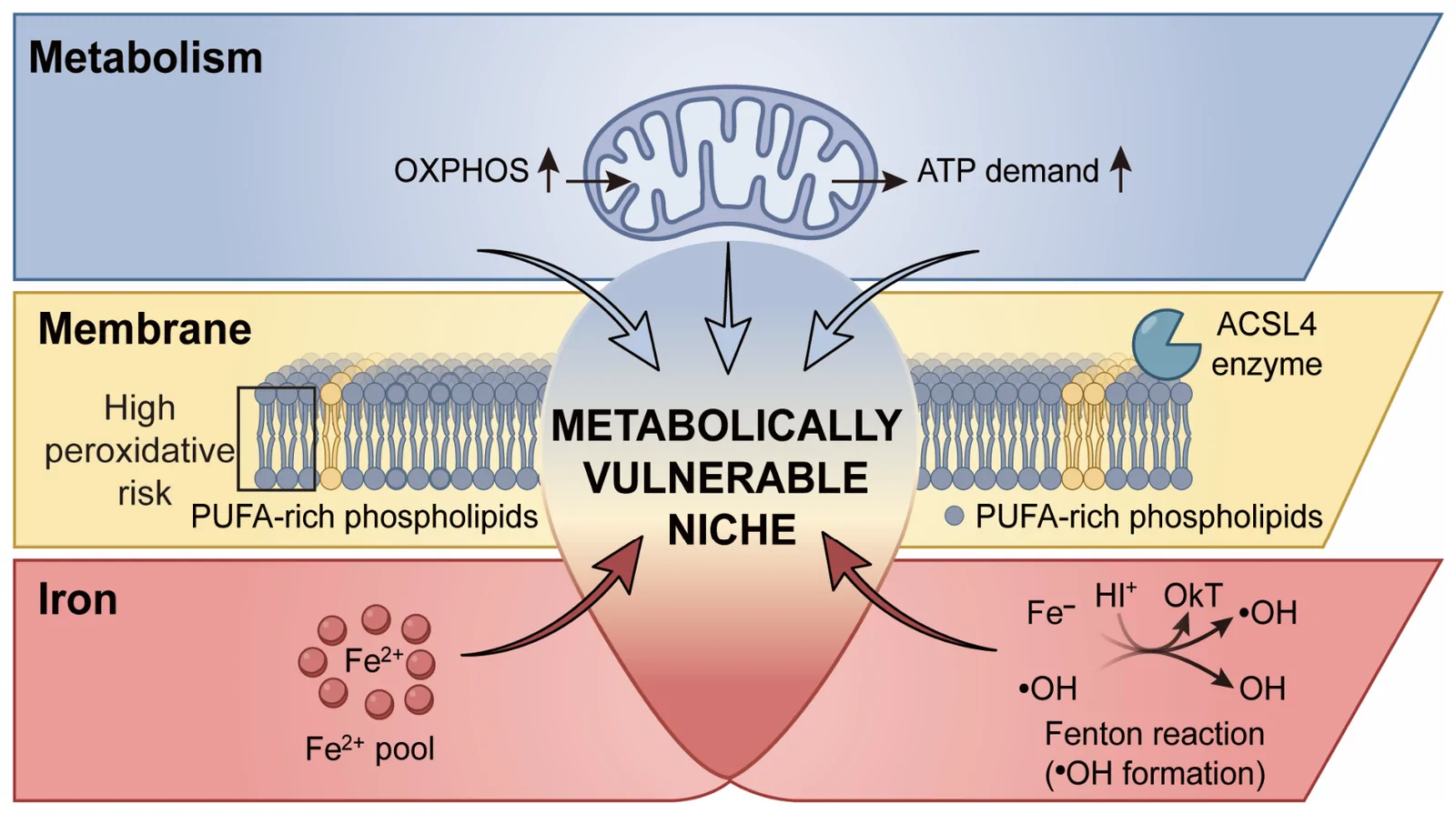
Core biological dimensions establishing a hypothesized ferroptosis-prone niche in the EUS. Upper (Metabolism): High OXPHOS and ATP demand are supported by EUS studies (D). Middle (Membrane): PUFA-rich phospholipid composition and ACSL4 activity are inferred from general striated muscle and cancer lipidomics (M/X). Lower (Iron): Labile Fe^2+^ accumulation and Fenton chemistry are extrapolated from I/R and hemorrhage models (X); direct EUS iron imaging is lacking. Upward arrows (↑) indicate an increase in the corresponding biological feature. (The figure was created using Adobe Illustrator 2020.)

Polyunsaturated fatty acids (PUFAs) are core structural components of striated muscle cell membranes, critical for maintaining membrane fluidity, supporting excitation-contraction coupling, and mediating mechanotransduction. This same chemical property, however, also increases susceptibility to lipid peroxidative stress [16,22]. A growing body of evidence indicates that cellular ferroptosis sensitivity can be substantially influenced by membrane lipid composition [16,17]. Striated muscle sarcolemma is naturally abundant in PUFA-containing phospholipids, which may account for a substantial proportion of membrane phospholipid composition (M; inferred from general skeletal muscle lipidomics [16,18]; direct EUS sarcolemma lipid profiling remains unavailable). This lipid profile supports the membrane flexibility and stability required for repeated contraction and mechanical loading, and is particularly relevant for the EUS, which must sustain basal tone and accommodate rapid mechanical stress fluctuations throughout life. This creates an evolutionary trade-off: the membrane composition that enables normal sphincter function simultaneously increases the tissue’s intrinsic vulnerability to lipid peroxidative injury (H). Membrane lipid composition is also heterogeneously distributed across subcellular compartments, creating distinct lipid microenvironments with variable susceptibility to oxidative modification and ferroptotic damage [23,24]. Notably, membrane lipid composition exhibits measurable plasticity (Figure 2) [25,26].

Under homeostatic conditions, the EUS relies on a multi-tiered antioxidant defense system to counteract the continuous ROS generated by mitochondrial respiration [27]. Antioxidant capacity is not a static property; it is dynamically modulated by aging, local ischemia, chronic mechanical stress, inflammation, and mitochondrial damage. A study of middle-aged rat urethral tissue demonstrated widespread downregulation of core antioxidant enzymes [28].

### 3.4. Exposure to Repeated Mechanical and Ischemic Stress

The unique anatomical position of the EUS subjects it to recurrent mechanical compression, causing transient but repeated cycles of microvascular occlusion and subsequent reperfusion. Among the diverse pathological stresses affecting pelvic floor function, ischemia–reperfusion (I/R) injury occupies a unique position as the bridge between mechanical tissue damage and metabolic injury. Microsphere perfusion studies have confirmed that vaginal distension drastically reduces blood flow to the bladder, urethra, and vaginal tissue. Histologically, the EUS sustains the most severe injury in this model (D, rat simulated childbirth) [29,30]. A defining feature of EUS I/R injury is its recurrent, cumulative nature. During the ischemic phase, oxygen deprivation stalls the mitochondrial electron transport chain, leading to accumulation of reduced electron carriers and disruption of redox homeostasis. During reperfusion, the sudden reintroduction of oxygen drives severe electron leakage and excessive reactive oxygen species production, which oxidizes membrane phospholipids and creates a ferroptosis-permissive environment. Concurrently, muscle fiber tearing, microvascular rupture, and local microhemorrhage release hemoglobin and myoglobin (D for EUS I/R injury; X for hemoglobin/myoglobin-derived iron toxicity, extrapolated from cardiac and skeletal muscle I/R models) [31,32].

**Figure 3:**
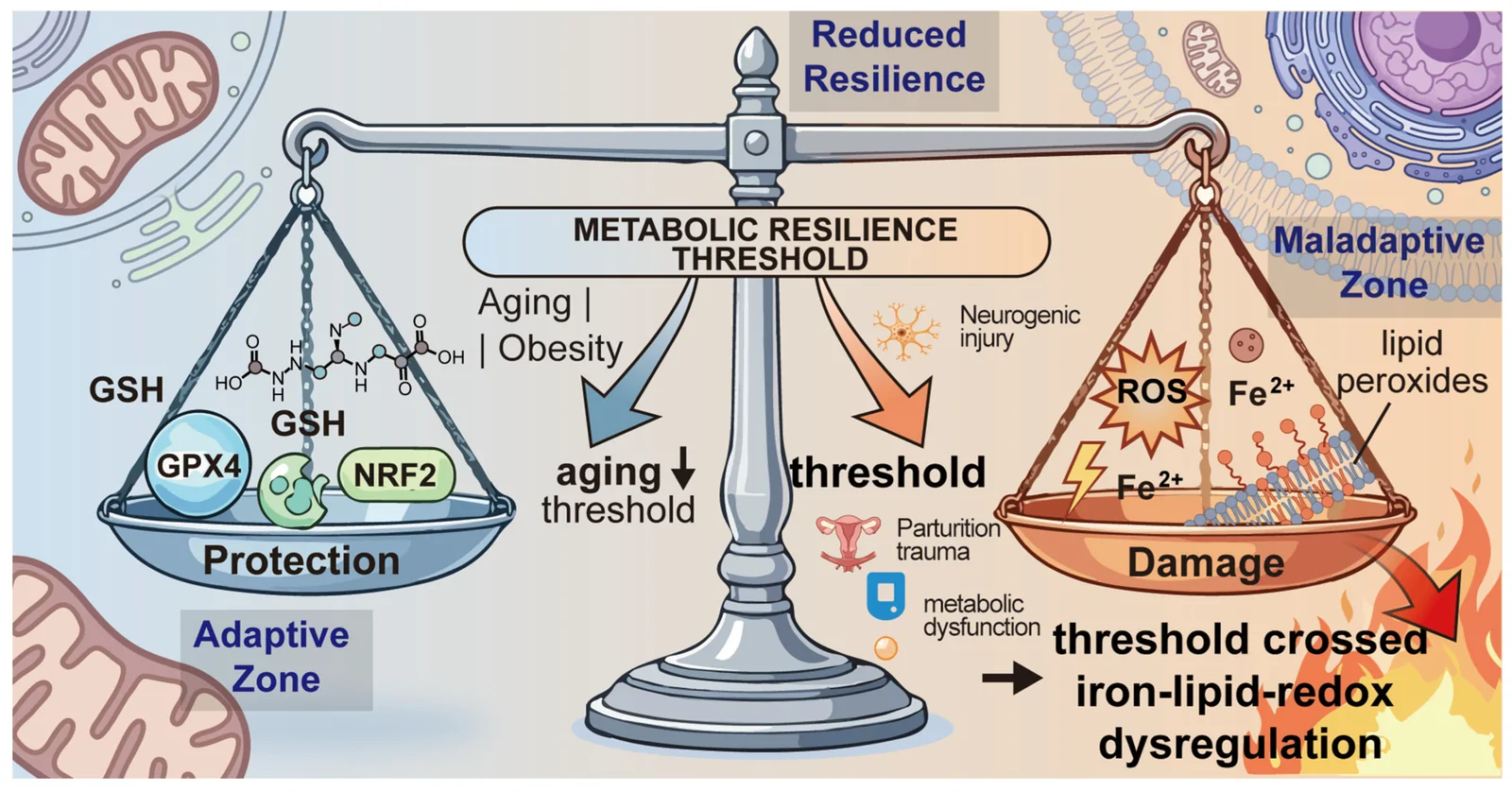
Conceptual model of a hypothesized EUS regenerative resilience threshold. The transition from the adaptive zone to the maladaptive zone is driven by cumulative metabolic and physical stressors. Importantly, this threshold is a conceptual framework representing the tipping point of tissue homeostasis, rather than a quantitatively defined biochemical parameter. Aging intrinsically lowers resilience, while clinical insults may overwhelm protective reserves and trigger ferroptosis-associated degeneration within the EUS niche. Arrows indicate the proposed direction of change or progression; downward arrows denote a reduction in the metabolic resilience threshold or progression toward a maladaptive state. (The figure was created using Adobe Illustrator 2020.)

A single I/R episode may only cause a temporary decrease in tissue resilience. Repeated I/R insults may progressively reduce this conceptual resilience reserve, potentially shifting the tissue toward a maladaptive low-resilience state in a maladaptive low-resilience state at baseline. This is the mechanistic core of the persistent degeneration paradox: patients do not experience continuous new injuries, but remain locked in a low-threshold pathological state long after the initial insult. The convergence of iron overload and severe oxidative stress during I/R is a well-documented driver of tissue damage across organ systems [33,34]. Synthesizing these four vulnerability axes allows us to formalize a conceptual model for EUS degeneration: the hypothesized metabolic resilience threshold. Collectively, these interrelated biological features contribute to the metabolic vulnerability of the EUS (Figure 3).

## 4. Clinical Insults Establish a Metabolic Vulnerability Landscape

The EUS shares core features of the striated muscle system: excitation-contraction coupling, dependence on mitochondrial oxidative metabolism, dynamic adaptation to mechanical load, and reliance on resident stem cells for tissue renewal. These upstream stressors do not act in isolation; they converge through interconnected signaling networks to collectively erode metabolic resilience, lower the conceptual threshold for oxidative damage, and potentially increase susceptibility to ferroptosis-associated stress. Clinically, four categories of insults represent the well-recognized contributors of EUS degeneration.

### 4.1. Parturition Trauma

Vaginal delivery represents the most intense physiological mechanical stress experienced by the female pelvic floor. During parturition, the EUS and its surrounding supportive structures undergo sustained compression, excessive stretch, and focal vascular occlusion [35]. As noted above, vaginal distension in animal models triggers a classic I/R injury cascade [29,30]. Local microhemorrhage and erythrocyte degradation release redox-active free iron, further promoting lipid peroxidation and creating a microenvironment highly permissive to ferroptosis-associated damage [36]. Beyond I/R injury, mechanical stretch itself directly modulates oxidative stress pathways [34]. The post-traumatic inflammatory response also contributes to long-term resilience erosion [37,38]. In aggregate, parturition trauma simultaneously induces mechanical injury, I/R stress, inflammatory activation, and iron homeostasis perturbation, potentially creating a microenvironment permissive to ferroptosis-associated stress that may erode baseline tissue resilience for decades after the initial injury.

### 4.2. Aging

Aging is the one of the strongest population-level risk factors for EUS functional decline and SUI. With advancing age, striated muscle tissues universally develop iron homeostasis dysregulation, mitochondrial dysfunction, and chronic low-grade inflammation [39,40,41,42,43]. Aged skeletal muscle exhibits an expanded labile iron pool, alongside defective mitochondrial quality control and accumulated mitochondrial DNA damage [44]. In the urethra specifically, antioxidant capacity declines markedly with age [28,45]. Skeletal muscle aging models recapitulate key ferroptosis-associated features: increased lipid peroxidation, mitochondrial shrinkage, and impaired GPX4 defense systems [46]. Concurrently, satellite cell regenerative capacity declines, and pro-fibrotic stromal phenotypes accumulate, further reducing tissue reparative ability [47,48].

### 4.3. Systemic Metabolic Dysfunction

Beyond discrete acute injuries, systemic metabolic abnormalities represent a pervasive, underrecognized modulator of local EUS vulnerability. Epidemiological data consistently link obesity, type 2 diabetes, and dyslipidemia to higher SUI incidence and poorer treatment response [49,50,51]. The high prevalence of metabolic disorders in aging populations amplifies this vulnerability [52,53,54,55]. Systemic metabolic dysfunction drives chronic low-grade inflammation, microvascular endothelial damage, and systemic oxidative stress, all of which permeate the local sphincter microenvironment. The core effect of systemic metabolic dysfunction is to lower this conceptual threshold, driving the local sphincter tissue to transition more easily from adaptive repair to degenerative decline. Dyslipidemia may promote accumulation of peroxidation-prone lipids with peroxidation-prone fatty acid species, while hyperglycemia augments mitochondrial ROS production and impairs antioxidant defense capacity [34]. Metabolic syndrome models further show widespread mitochondrial damage, reduced ATP levels, and decreased nerve density in lower urinary tract tissues [56]. These findings position systemic metabolic dysfunction not as a primary initiator of EUS damage, but as a critical vulnerability factor that lowers this conceptual resilience threshold, thereby amplifying the impact of mechanical, ischemic, and age-related stressors.

### 4.4. Neurogenic Injury

EUS function is entirely dependent on intact pudendal nerve innervation [57]. Nerve injury not only abolishes evoked contractile function, but triggers extensive metabolic reprogramming within the muscle tissue [58,59]. Denervated muscle also shows reduced GPX4 activity and impaired glutathione utilization, indicating disruption of the core ferroptosis defense system [60]. Concurrently, denervation triggers aberrant activation of fibro-adipogenic progenitors (FAPs), macrophages, and satellite cells [61]. In recent years, extracellular vesicle (EV)-mediated intercellular communication has emerged as a potential mechanism for propagating stress signals across the niche [62,63,64,65,66]. Taken together, diverse pathological triggers—acting across acute, chronic, and systemic timescales—converge on a shared set of metabolic disruptions in the EUS niche. The key clinical phenotypes, their corresponding ferroptosis-associated molecular signatures, and the supporting evidence are summarized in Table 1.

## 5. Iron–Lipid–Redox Dysregulation Converts Metabolic Vulnerability into Biochemical Degeneration

**Figure 4:**
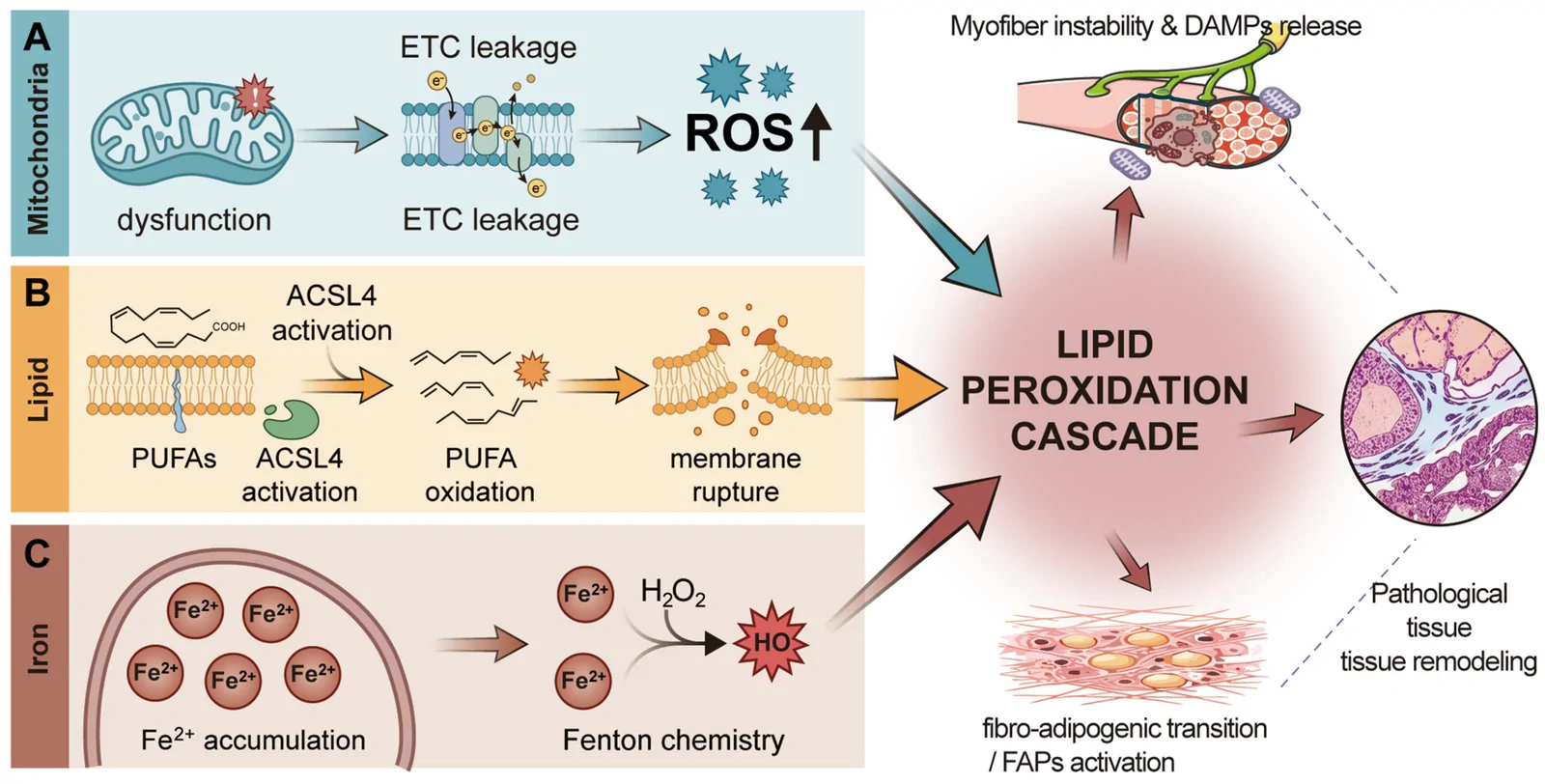
Core regulatory modules of ferroptosis-mediated biochemical degeneration, as inferred from skeletal muscle and cross-tissue models (M/X). Direct validation of this exact cascade in EUS tissue is not yet available. (**A**) Mitochondrial dysfunction and ETC leakage are documented in pelvic floor and muscle aging models (M/D). (**B**) ACSL4-driven PUFA oxidation and membrane rupture are established in cancer/hepatic models (X); EUS-specific ACSL4 activity is unknown. (**C**) Fe^2+^ accumulation and Fenton chemistry are extrapolated from I/R and hemorrhage models (X). ! denotes cellular or subcellular stress/damage; ↑ indicates an increase or upregulation of the corresponding biological feature. (The figure was created using Adobe Illustrator 2020.)

It is within this metabolically constrained landscape that ferroptosis emerges as a potential molecular integrator. Ferroptosis is a unique form of regulated cell death driven by iron-dependent lipid peroxidation, mechanistically distinct from apoptosis, necrosis, and autophagy [13,14,15]. The primary defense against this form of oxidative destruction is the System Xc^−^–GSH–GPX4 antioxidant axis [67,68]. Recent studies in skeletal muscle biology suggest that ferroptosis-associated metabolic disturbances may extend beyond terminal cell death and influence tissue repair processes [41,69,70,71,72]. Therefore, we propose that ferroptosis-associated stress should be considered a potential molecular integrator within the broader metabolic vulnerability framework, rather than an isolated mechanism of acute cell death. A growing body of research indicates that the pathophysiological significance of ferroptosis extends far beyond executing terminal cell death. More importantly, it provides a mechanistic link connecting dysregulated iron metabolism, progressive lipid peroxidation, and microenvironmental remodeling during tissue degeneration. While direct evidence specific to the EUS remains limited, available studies indicate that multiple SUI-associated stressors converge on three core regulatory modules (Figure 4). In this capacity, this iron–lipid–redox dysregulation functions not as a singular causal agent, but as a molecular integrator that translates diverse metabolic stress signals into a unified biochemical vulnerability. The mechanistic modules in this section integrate evidence spanning multiple tissue types. We flag each pathway according to its current evidentiary status in the EUS niche: direct (observed in EUS/SUI models), muscle-extrapolated (observed in other skeletal muscle), cross-tissue (observed in cardiac/hepatic/cancer models), or hypothetical (inferred from general redox biology). This framework clarifies which components are empirically anchored and which remain testable hypotheses.

### 5.1. Iron Dysregulation

Iron is an essential cofactor for mitochondrial respiration, oxygen transport, and the catalytic activity of dozens of metabolic enzymes. Ferritinophagy, the selective autophagic degradation of ferritin mediated by nuclear receptor coactivator 4 (NCOA4), is a core regulatory mechanism of cellular iron cycling (X; established in hepatic, cardiac, and cancer models [73]; EUS ferritinophagy activity uncharacterized). In the EUS niche, local microhemorrhage, post-injury hemoglobin degradation, and age-related iron metabolism redistribution all disrupt local iron homeostasis (H; inferred from EUS exposure to I/R and microhemorrhage, but direct quantification of labile Fe^2+^ in EUS tissue is lacking). Critically, ferrous iron (Fe^2+^) directly catalyzes the Fenton reaction to generate hydroxyl radicals, whose aberrant accumulation further drives lipid peroxidation and oxidative damage propagation. Across skeletal muscle disease models, dysregulation of the iron metabolic network is consistently linked to tissue degeneration [31]. Mechanical stress, I/R injury, and microcirculatory dysfunction further amplify the impact of iron dysregulation on tissue integrity [74]. Interventional studies with iron chelators reinforce the functional relevance of iron homeostasis [75,76]. Dysregulated iron homeostasis may therefore represent an important upstream contributor to the establishment of a ferroptosis-permissive biochemical environment in the EUS.

### 5.2. System Xc^−^–GSH–GPX4 Failure

If dysregulated iron homeostasis provides the chemical prerequisite for lipid peroxidation, cellular antioxidant defense capacity determines whether the tissue can contain oxidative damage and prevent cascade propagation. Among all known ferroptosis regulatory mechanisms, the System Xc^−^–glutathione (GSH)–glutathione peroxidase 4 (GPX4) axis is recognized as a central endogenous protective system against lipid peroxidative stress. System Xc^−^, a heterodimer of SLC7A11 and SLC3A2, mediates extracellular cystine uptake to supply the rate-limiting substrate for GSH synthesis. GPX4 then uses GSH as an electron donor to reduce toxic lipid hydroperoxides to inert lipid alcohols [77]. Impairment of any component of this axis increases cellular ferroptosis susceptibility: GSH depletion [14], reduced SLC7A11 expression [78], GPX4 inactivation [67], and dysfunction of the NADPH-dependent reductive cycle [15] all sensitize cells to ferroptosis-related metabolic failure. Aging, ischemia, chronic inflammation, and metabolic dysfunction all compromise this defense network, progressively eroding its protective capacity. Nrf2 is a central transcriptional regulator of this defense system [79]. Age-related decline in Nrf2 activity is a well-documented driver of reduced tissue antioxidant capacity [27]. In SUI rat models, sulforaphane (SFN) activates the Nrf2-ARE pathway, improves urethral function, prolongs micturition intervals, and increases peak voiding pressure, while elevating SOD, GSH-Px, and CAT activity [80].

### 5.3. Lipid Peroxidation and Membrane Vulnerability

Lipid peroxidation is the defining biochemical event of ferroptosis. Initiated either by free iron-catalyzed Fenton reactions or lipoxygenase (LOX)-mediated enzymatic oxidation, PUFAs in membrane phospholipids undergo sustained oxidative attack, leading to progressive accumulation of lipid peroxidation products and the biochemical compromise of membrane architecture. Lipid peroxidation does not occur uniformly across the cell membrane. Distinct subcellular compartments differ markedly in lipid composition, iron distribution, and redox status. In pelvic floor muscle models, mechanical stretch induces 15-LOX-1 upregulation alongside GPX4 downregulation and ferroptosis-like phenotypic changes (D for mouse pelvic floor muscle stretch) [34], suggesting LOX-mediated lipid oxidation participates in the pathological response to mechanical injury. ACSL4-mediated PUFA membrane incorporation is also an important contributor to ferroptosis susceptibility (X; robust in cancer and hepatic models; no EUS-specific ACSL4 expression or lipidomic data) [16,17]. At the molecular level, the terminal lipid peroxidation products (such as MDA and 4-HNE) profoundly alter membrane biophysical properties, setting the stage for downstream structural failure.

### 5.4. Ferroptosis-Associated Stress as a Regenerative Failure Switch

The most profound implication of viewing SUI through a metabolic vulnerability lens is that ferroptosis-associated biochemical alterations may not only compromise mature EUS myofibers but may also impair the tissue’s regenerative machinery. Importantly, ferroptosis-associated damage does not necessarily require complete execution of cell death. Sublethal lipid peroxidation stress may be sufficient to disrupt cellular metabolism, impair differentiation programs, and reshape the regenerative niche [81,82]. As established in the resilience threshold model, successful EUS repair relies on the activation, proliferation, and fusion of resident satellite cells. However, emerging evidence in skeletal muscle biology suggests that a ferroptosis-permissive microenvironment may fundamentally disrupt this regenerative cascade. Resident satellite cells are highly sensitive to their local redox environment. While transient, physiological ROS signaling is required for initial satellite cell activation, chronic exposure to iron overload and lipid peroxidation induces severe stem cell dysfunction [83,84]. In models of muscular dystrophy and sarcopenia, disrupted iron homeostasis and GPX4 depletion have been associated with impaired satellite cell maintenance and reduced regenerative capacity, and may ultimately halt the myogenic differentiation program (M; muscular dystrophy and sarcopenia models [70,71]; direct EUS satellite cell ferroptosis not demonstrated). Furthermore, myofibers undergoing ferroptosis-associated stress may release distinct damage-associated molecular patterns (DAMPs) that may substantially alter local immune signaling. During normal muscle regeneration, local macrophages transition from a pro-inflammatory (M1-like) state that clears debris to an anti-inflammatory (M2-like) state that supports myogenesis. However, the lipid peroxides and inflammatory mediators released during ferroptotic stress may maintain macrophages in a prolonged pro-inflammatory state. This derailed immune-metabolic crosstalk may inhibit myogenic fusion and instead may stimulate adjacent fibro-adipogenic progenitors (FAPs) to deposit extracellular matrix. Thus, sublethal ferroptotic stress and lipid peroxidation may act as a candidate molecular link explaining the persistent degeneration paradox: by potentially exhausting satellite cell reserves and skewing the microenvironment toward fibro-adipogenic remodeling, ferroptosis-associated signaling may prevent the injured sphincter from regenerating, potentially locking the EUS in a state of chronic functional incompetence.

## 6. Ferroptosis-Related Metabolic Vulnerability and Regenerative Failure Determine EUS Fate

EUS structural integrity depends not only on long-term myofiber survival but also on the sustained regenerative capacity of the resident niche. Within the proposed framework, persistent metabolic and redox stress may progressively shift the tissue from effective repair toward regenerative insufficiency and fibrotic remodeling. Ferroptosis-associated signaling is considered a candidate biochemical mechanism within this transition, potentially affecting both mature myofibers and regenerative cell populations.

### 6.1. Myofiber Instability

Mature myofibers are the primary functional units of the EUS, and their sustained viability is essential for contractile function. The progressive accumulation of lipid peroxidation products is hypothesized to translate into severe functional deficits, impairing excitation-contraction coupling and compromising the structural integrity of the contractile apparatus. Crucially, as these damaged myofibers progressively develop structural instability, they release large quantities of damage-associated molecular patterns (DAMPs), oxidized lipids, and inflammatory mediators [15]. This massive release of stress signals profoundly reshapes the local microenvironment, exposing adjacent regenerative stem cells to a toxic, pro-inflammatory milieu that further hinders adaptive repair.

### 6.2. Satellite Cell Exhaustion

Following injury, satellite cells activate from quiescence and undergo sequential proliferation, differentiation, and self-renewal to regenerate myofibers and repair tissue [85]. Successful regeneration requires both surviving mature myofibers and an intact satellite cell pool. Accumulating evidence indicates that persistent oxidative stress, mitochondrial dysfunction, and iron dysregulation can compromise satellite cell regenerative capacity across muscle systems [70,86,87]. Rather than attributing this effect to a single molecular pathway, these findings suggest that disruption of the iron–redox–lipid axis may impair the ability of regenerative cells to adapt to oxidative stress and progress through appropriate repair states. Thus, both iron overload and functional iron deficiency may compromise regeneration, while altered redox buffering may further constrain satellite cell proliferation and differentiation. Evidence for satellite cells within the human EUS supports their potential relevance to sphincter repair [8], but their activation trajectory, regenerative capacity, and susceptibility to ferroptosis-associated stress after pelvic floor injury remain largely uncharacterized [88,89]. Accordingly, ferroptosis-associated signaling is best regarded as a candidate mechanism linking metabolic stress to impaired EUS regenerative reserve rather than an established driver of satellite cell exhaustion.

### 6.3. Fibro-Adipogenic Transition

Degenerative changes in the urethral sphincter manifest as progressive loss of contractile myofibers, accompanied by increased extracellular matrix deposition, collagen hyperplasia, and reduced tissue compliance [90,91,92]. When ferroptosis-associated stress contributes to progressive myofiber loss while simultaneously impairing satellite cell-mediated regeneration, the tissue may be unable to achieve effective functional repair and may be ultimately diverted toward a fibrotic replacement pathway, forming a self-reinforcing degenerative cycle. In striated muscle tissue, fibro-adipogenic progenitors (FAPs) are key regulators of tissue remodeling. Under physiological conditions, FAPs support muscle regeneration and maintain tissue homeostasis; under chronic inflammation and persistent injury, however, FAPs undergo pathological activation and shift toward pro-fibrotic and adipogenic differentiation, leading to collagen deposition and scar formation [93,94]. Accumulating evidence indicates that ferroptosis-associated oxidative stress, lipid peroxidation products, and DAMP-mediated inflammatory signaling may contribute to this pathological transition [61,95]. Once fibrotic replacement becomes the dominant repair mode, however, tissue stiffness increases markedly, regenerative potential is depleted, and the system becomes trapped in a stable degenerative state (Table 2).

## 7. Evidence Hierarchy, Limitations, and Unresolved Questions

### 7.1. Evidence Hierarchy and Extrapolation Limits

The framework advanced in this review draws on evidence that spans human EUS biology, comparative striated muscle physiology, and cross-tissue redox research. Such breadth is necessary because direct mechanistic studies of ferroptosis in the native human EUS remain scarce; yet this same breadth introduces a fundamental interpretive challenge. We have therefore classified the evidence underpinning each mechanistic claim according to its proximity to the EUS niche: direct observation in EUS tissue or validated injury models (D), replication in other skeletal muscle systems (M), observation in non-muscle organs such as heart, liver, or cancer cell lines (X), and inference from general biochemical principles (H).

When viewed through this lens, a clear gradient emerges. The metabolic preconditions for ferroptosis susceptibility—continuous OXPHOS, PUFA-enriched membranes, and recurrent I/R exposure—are anchored in EUS physiology (D). However, the execution machinery that converts these preconditions into cell death or regenerative failure rests on shakier ground. Iron dyshomeostasis and ferritinophagy have not been directly profiled in EUS myofibers; they are inferred from cardiac I/R and hepatic iron overload studies (X) and from aged limb muscle (M). Similarly, while Nrf2 activation improves urethral function in SUI rats (D), whether this benefit operates specifically through ferroptosis suppression—as opposed to general antioxidant upregulation—remains unclear (M/X). ACSL4-driven lipid remodeling in EUS membranes remains to be experimentally validated (H), and the satellite-cell dysfunction we describe is extrapolated from non-EUS muscle injury, dystrophy, and disuse atrophy models (M). Table 1 and Table 2 summarize these distinctions.

Consequently, the systemic metabolic vulnerability signature characterized by ferroptosis-associated stress should be viewed as a conceptual framework that integrates interdisciplinary findings and proposes testable mechanisms underlying EUS regenerative failure. Although direct causal validation remains necessary, this framework provides a structured basis for investigating how metabolic stress may influence tissue resilience and degeneration.

### 7.2. Alternative and Complementary Mechanisms

Treating ferroptosis-associated stress as the exclusive driver of EUS degeneration would oversimplify the biological complexity of the sphincter niche. Several parallel processes may independently erode regenerative resilience, and their relationship to iron-dependent lipid peroxidation is likely one of co-existence and mutual reinforcement rather than simple subordination.

Cellular senescence offers a particularly relevant example. Aging and repeated mechanical injury can push satellite cells into a senescent state characterized by cell-cycle arrest and SASP-mediated inflammation [96,97]. This process may impair regeneration without requiring ferroptotic execution; conversely, the chronic inflammatory milieu created by senescent cells may heighten local iron availability and lipid peroxidation, thereby lowering the threshold for ferroptosis-associated damage. The two pathways are thus bi-directionally permissive rather than hierarchically nested.

ECM remodeling presents another independent axis. Collagen deposition, crosslinking, and tissue stiffening directly compromise force transmission and sphincter compliance, and these structural changes can proceed through TGF-β-driven fibroblast activation without prior ferroptotic myofiber death [98]. In this scenario, ferroptosis may accelerate myofiber loss and secondarily trigger FAP activation, but it is not the only route to the fibrotic endpoint that characterizes advanced EUS degeneration.

Hormonal transitions, particularly estrogen deficiency after menopause, constitute a distinct upstream modifier. Epidemiological data firmly link menopausal status to SUI incidence and progression yet remains insufficiently characterized. Estrogen influences both muscle fiber type composition and connective tissue remodeling; its decline may therefore operate through parallel channels that converge on regenerative failure without necessarily passing through an iron–lipid–redox checkpoint [99].

Finally, other regulated cell death modalities—including apoptosis, necroptosis, pyroptosis, and autophagic dysfunction—have each been implicated in striated muscle degeneration across diverse disease contexts [100]. None of these pathways should be regarded as mutually exclusive with ferroptosis. In chronic degenerative conditions, it is increasingly recognized that multiple cell death programs can coexist, sequentially activate, or even cross-amplify. A myofiber undergoing necroptotic swelling may release DAMPs that trigger local inflammation, which in turn depletes GSH and sensitizes neighboring cells to ferroptosis; alternatively, sublethal lipid peroxidation may commit a satellite cell to senescence rather than death [100].

What emerges from this complexity is not a single causal chain, but a network in which ferroptosis-associated metabolic stress occupies one plausible convergence node. It integrates iron dyshomeostasis, lipid peroxidation, and antioxidant collapse into a coherent biochemical signature, yet it does so alongside senescent, inflammatory, and ECM-remodeling pathways that may initiate independently, interact dynamically, and collectively determine whether the EUS retains or loses its capacity for functional repair.

### 7.3. Cellular Heterogeneity and Niche Complexity

Beyond the absence of human histological data, the cellular complexity of the EUS niche remains poorly resolved. Mature myofibers, satellite cells, FAPs, vascular endothelial cells, immune cells, and nerve-associated cells collectively form the sphincter microenvironment, and distinct cell populations may have vastly different ferroptosis susceptibility and stress response profiles. The absence of cell type-specific evidence is a major barrier to mechanistic dissection, as it remains unknown whether ferroptotic damage initiates predominantly in mature myofibers, preferentially depletes the satellite cell pool, or primarily activates FAPs.

Two non-mutually exclusive initiating scenarios may explain this cellular origin problem. Under a myofiber-first scenario, mechanical injury triggers metabolic stress and mitochondrial dysfunction within mature contractile fibers, leading to lipid peroxidation and DAMP release that secondarily poison the regenerative niche. Under a niche-first scenario, vascular rarefaction, denervation, or interstitial inflammation alter the metabolic microenvironment before significant myofiber death occurs, causing satellite cell dysfunction that subsequently leaves myofibers without replacement capacity [97,101]. Discriminating between these models should be a priority for future EUS-specific studies. If the myofiber-first model predominates, early biomarkers should reveal myofiber-specific lipid peroxidation and GPX4 loss preceding satellite cell depletion; if the niche-first model holds, endothelial dysfunction or denervation markers should appear before measurable myofiber instability.

### 7.4. Temporal Sequence and State-Dependent Defense

A third constraint involves the unresolved temporal sequence of pathological events. It remains unclear whether ferroptosis represents an early initiating event, a transitional checkpoint, or a late consequence of chronic degeneration. Mechanical injury, ischemia–reperfusion, neurogenic damage, and chronic inflammation all induce oxidative stress, which in turn may promote ferroptosis. The two most likely form a mutually reinforcing feedback network, and their causal relationship requires clarification through dynamic tracking studies rather than static cross-sectional observations.

Equally important is the state-dependent nature of antioxidant defense networks. Observed downregulation of certain antioxidant factors—such as xCT/SLC7A11 during satellite cell differentiation—does not necessarily indicate pathological collapse; it may reflect physiological regulation at specific developmental stages [102,103]. Future research must therefore carefully distinguish adaptive remodeling from pathological dysregulation, as misinterpreting physiological redox shifts as therapeutic targets could lead to counterproductive interventions.

### 7.5. Model Translation Barriers

Model translation presents a fifth barrier. Most existing studies use rodent models or in vitro cultures, which cannot fully recapitulate the neuromuscular junction, vascular network, and complex chronic mechanical stress environment of the native human EUS. The anatomical and physiological differences between rodent pelvic floor muscles and the human EUS—particularly regarding fiber type distribution, innervation density, and mechanical loading patterns—limit the direct extrapolation of preclinical findings to clinical populations [104,105].

A related translational challenge is the incomplete characterization of the biological determinants underlying differential sphincter recovery in patients. A recent systematic review and meta-analysis of fixed sling implantation for postprostatectomy stress urinary incontinence identified multiple patient- and disease-related factors associated with treatment failure, highlighting substantial heterogeneity in clinical outcomes even within a relatively standardized sphincter-supporting intervention [106]. Although these findings derive from male postprostatectomy incontinence and cannot be directly extrapolated to female SUI or the EUS, they underscore the broader clinical challenge of explaining why apparently similar sphincter dysfunction may lead to different functional outcomes. Future studies should therefore integrate longitudinal clinical phenotyping with molecular and functional assessment of EUS tissue to determine whether differences in metabolic resilience, regenerative reserve, or redox vulnerability contribute to divergent recovery trajectories.

### 7.6. Lipid Metabolic Remodeling

Finally, current understanding of lipid metabolic remodeling remains preliminary. Systematic investigations of EUS tissue lipidomic profiles, oxidized phospholipid distribution patterns, and spatial localization of key enzymes such as ACSL4 and LOXs are lacking. Inferences regarding abnormal lipid metabolism promoting ferroptosis in the EUS therefore remain largely theoretical, and lipid-centric therapeutic hypotheses await organ-specific lipidomic validation [107,108].

**Table 2:** Candidate Iron–Lipid–Redox Mechanisms Associated with the EUS Repair-to-Degeneration Transition.

Checkpoint	Core Components	Biochemical Effect	Pathological Consequence	Cross-Talk	Evidence
Iron homeostasis	NCOA4, FTH1, TFR1, FPN, HMOX1	Ferritinophagy ↑ → labile Fe^2+^ pool; Fenton reaction → •OH; disrupts mitochondrial recovery	Lipid peroxidation initiation → membrane erosion → impaired E-C coupling → ferroptosis-associated structural compromise; I/R exacerbates iron overload via Hb/Mb degradation	Substrate for Membrane checkpoint (Fenton); overwhelms Redox checkpoint (GSH depletion); iron overload in Satellite cells impairs Stem cell checkpoint via RNF20/H2Bub1	[31,73,74,75,76]
Redox buffering	SLC7A11/SLC3A2, GPX4, GSH, NADPH; Nrf2, ARE	Cystine uptake impairment → GSH depletion → GPX4 disability; Nrf2 maintains antioxidant/iron-homeostatic programs	Toxic phospholipid oxidation → catastrophic membrane injury → E-C coupling loss; progressive redox buffering erosion	Permits Iron checkpoint Fenton chemistry; GSH depletion sensitizes Membrane checkpoint; redox collapse in Satellite cells arrests Stem cell checkpoint	[14,15,27,67,77,78,79,80]
Membrane vulnerability	ACSL4, LOX (15-LOX-1), PUFA-PLs	ACSL4-mediated oxidizable PUFA-PL enrichment; LOX enzymatic oxidation + Fenton chemistry initiate peroxidation	MDA/4-HNE alter membrane biophysics → structural compromise; mitochondria-associated membranes and lipid rafts as preferential vulnerability sites	Lipid peroxidation products (4-HNE/MDA) as DAMPs activate Stem cell checkpoint (FAP stimulation, M1 lock); depletes GSH, stressing Redox checkpoint	[16,17,34]
Stem cell–stromal	Satellite cells (SCs; PAX7, MyoD, MyoG), TFR1, GPX4; FAPs, TGF-β	Iron overload + lipid peroxidation disrupt Satellite cell activation/self-renewal; ferroptotic myofibers release DAMPs/oxidized lipids → M1 lock + FAP stimulation	MuSC depletion → impaired myofiber replacement; FAP activation → collagen deposition + fat infiltration → fibrotic replacement → reduced compliance	Satellite cell dysfunction removes brake on Membrane-driven fibrosis; FAP-derived TGF-β suppresses MuSC activation; iron dyshomeostasis in stem cells links to Iron checkpoint	[61,70,81,82,83,84,87,93,94,95,109]

Evidence classification: Direct EUS evidence indicates observation in human EUS tissue, primary EUS myoblasts (US2KD line), or validated animal EUS injury models (simulated vaginal delivery, pudendal nerve injury, etc.). The table highlights that while upstream EUS vulnerabilities (high OXPHOS, PUFA-rich membranes, recurrent I/R) are empirically documented (D), the iron–lipid–redox dysregulation machinery—iron dyshomeostasis, GPX4 collapse, ACSL4-driven peroxidation, and DAMP-mediated FAP activation—currently relies on extrapolation from non-EUS systems (M/X). ↑ indicate an increase in the corresponding biological feature.

## 8. Restoring Metabolic Resilience: Biomarkers and Therapeutic Strategies

A critical implication of the metabolic vulnerability framework is the conceptual existence of a hypothesized reversible window. Rather than an already established clinical timeframe, this window represents a testable hypothesis to guide future translational research. We propose defining and capturing this window through three interconnected levels:

Biological definition: Biologically, this window represents an early-to-intermediate pathological stage where the EUS microenvironment is actively burdened by lipid peroxidation, mitochondrial dysfunction, oxidative stress, inflammatory activation, and satellite-cell dysfunction. Crucially, however, terminal fibrosis and adipogenic replacement remain limited. Before the system fully crosses this conceptual resilience threshold, the tissue retains sufficient biological plasticity, meaning that metabolic reprogramming or cell therapy might still restore the EUS to a regenerative homeostatic state [110].

Candidate surrogate markers: At the level of early detection, current clinical assessments rely on late-stage urodynamic symptoms that lack molecular sensitivity [111,112]. To clinically capture this hypothesized window, future diagnostics must identify candidate surrogate markers rather than relying on definitive, single-molecule diagnoses. Potential targets for non-invasive profiling include circulating or urinary oxidized lipid products, specific 4-HNE/MDA-related signatures [113], local inflammatory markers, and extracellular vesicles (EVs) encapsulating distinct metabolic signatures [114,115].

Potential imaging approaches: In parallel, advanced non-invasive imaging modalities represent potential future approaches to detect these tissue-level structural and metabolic shifts. Modalities such as high-resolution pelvic floor magnetic resonance imaging (MRI), diffusion-weighted MRI, T2 mapping, ultrasound-based muscle morphology, and elastography could theoretically be adapted to evaluate EUS tissue composition, hydration, and compliance dynamically [111,112], identifying the vulnerable niche before irreversible fibrotic remodeling dominates.

As research into ferroptosis as a modulator of tissue injury and repair homeostasis advances, its diagnostic and therapeutic value in pelvic floor dysfunction has attracted growing attention. Translational efforts should focus not only on direct ferroptosis inhibition, but more broadly on restoring metabolic resilience, preserving regenerative capacity, and redirecting the tissue repair trajectory away from fibrotic replacement (Table 3).

At the level of early detection, current clinical assessment of urethral sphincter dysfunction relies primarily on symptom scales, urodynamic testing, and imaging modalities [111,112]. While these approaches reflect anatomical structure, they lack sufficient sensitivity to detect molecular damage and changes in regenerative capacity within the sphincter microenvironment. Oxidative and lipid stress markers: Lipid peroxidation products [111,112] (MDA, 4-HNE) are widely used indicators of ferroptosis-associated metabolic stress [113]. However, systemic levels lack tissue specificity. Future diagnostics must pivot toward targeted profiling. Iron homeostasis markers: Circulating and local iron metabolism indices (serum ferritin, TfR1, HMOX1) may serve as surrogate markers of tissue iron dyshomeostasis [117]. Regenerative capacity markers: Markers reflecting satellite cell function and myogenic differentiation potential (PAX7, MyoD, MYOG) may provide insight into the intrinsic regenerative reserve [87]. Fibrotic remodeling markers: Extracellular matrix components (COL1A1, fibronectin) are established markers of advanced fibrotic transition [91]. In recent years, extracellular vesicles (EVs) have emerged as a promising bridge between the tissue microenvironment and liquid biopsy, with the potential to carry molecular signatures (microRNAs, oxidized lipids, and proteins) reflecting local redox status and ferroptotic activity [114,115].

At the level of endogenous metabolic restoration, interventions targeting the root of metabolic vulnerability aim to enhance adaptive capacity and improve mitochondrial function. The Nrf2 signaling pathway is a core regulator of endogenous antioxidant defense [80]. In SUI rat models, sulforaphane (SFN) activates the Nrf2-ARE pathway, improving urethral function and elevating SOD, GSH-Px, and CAT activity (D, rat SUI model) [80]. Local metabolic milieu optimization, such as physiological low-glucose culture or 3-hydroxybutyrate (3HB) treatment, further enhances autophagic and organellar remodeling capacity in human EUS myoblasts [110]. These approaches do not merely suppress pathology but actively rebuild the tissue’s intrinsic buffering capacity against oxidative insult.

**Figure 5:**
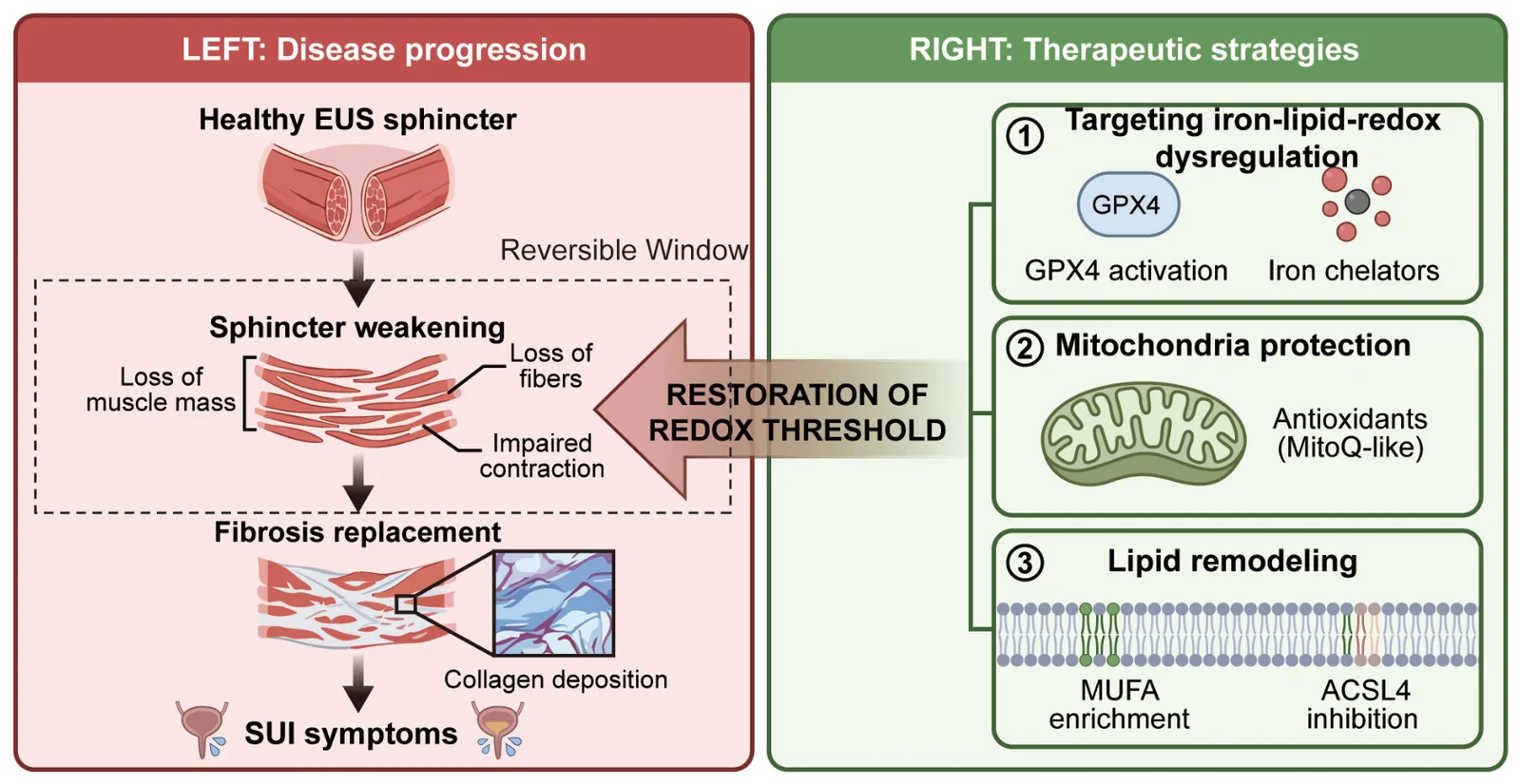
Disease progression trajectory and conceptual therapeutic strategies for restoring EUS metabolic resilience. **Left**: The proposed “reversible window” represents a testable hypothesis rather than a clinically validated stage. **Right**: A tiered therapeutic framework is proposed to progressively restore EUS regenerative resilience. Tier 1 aims to increase baseline resilience through metabolic optimization and cytoprotective strategies (e.g., sulforaphane [SFN] and 3-hydroxybutyrate [3HB]); Tier 2 aims to limit metabolic and redox amplification during acute or chronic stress (e.g., deferoxamine [DFO] and ferrostatin-1 [Fer-1]); and Tier 3 aims to restore the regenerative niche through cell-based approaches, extracellular vesicles (EVs), or biomaterials. The evidentiary basis varies across interventions: selected Tier 1 approaches have direct EUS/SUI model support (D), whereas Tier 2 and Tier 3 strategies are supported by pelvic floor, EUS injury, skeletal muscle, or related experimental models (D/M). This framework represents a proposed therapeutic sequence rather than a validated treatment algorithm; efficacy, optimal timing, and the potential need for combination or sequential intervention remain experimentally undetermined. (The figure was created using Adobe Illustrator 2020.)

For tissues already under significant oxidative stress, targeted inhibition of ferroptotic cascade amplification can preserve functional reserve. The iron chelator deferoxamine exerts tissue-protective effects by reducing the labile iron pool (M/X; skeletal muscle atrophy and postischemic protection [75]; not tested in EUS-specific ferroptosis models). Ferrostatin-1 has shown protective effects in mechanical stretch-induced pelvic floor muscle models by reducing lipid peroxidation and partially restoring ferroptosis-related molecular alterations (D, mouse pelvic floor muscle and C2C12 myoblasts) [34]. Such interventions should not be viewed as isolated treatments, but rather as components of a stage-oriented conceptual therapeutic framework spanning endogenous metabolic optimization (Tier 1), blockade of redox and metabolic amplification (Tier 2), and restoration of the regenerative niche (Tier 3), with the overarching goal of restoring EUS regenerative resilience and function (Figure 5).

Finally, at the level of regenerative niche reconstruction, merely inhibiting cell death is insufficient to fully restore tissue function. Cell therapy and engineered extracellular vesicle delivery systems aim to rebuild functional tissue architecture. VEGF-overexpressing mesoangioblasts promote EUS neuromuscular regeneration in simulated vaginal delivery models [4]. Adipose-derived stem cell extracellular vesicles combined with PEG hydrogel upregulate Nrf2/GPX4 axes, alleviate lipid peroxidation, and promote M2 macrophage polarization [116]. Modulating epigenetic programs to enhance antioxidant defense represents another emerging frontier for future sphincter degeneration intervention. These strategies shift the therapeutic goal from solely attenuating cell death-mediated damage to restoring the adaptive plasticity of the EUS microenvironment.

## 9. Future Perspectives

Whether a systemic metabolic vulnerability signature genuinely drives sphincter degeneration in human EUS tissue remains the foundational question. A direct chain of histological evidence must be established through transmission electron microscopy, localized lipid peroxidation assays, and molecular analysis of iron homeostasis regulators including GPX4, SLC7A11, ACSL4, NCOA4, and TFR1, evaluated in EUS tissue under conditions of parturition injury or aging. Complementary spatial multi-omics and iron imaging will further strengthen direct evidence of ferroptotic activity in the native human microenvironment.

How systemic metabolic vulnerability can be translated into clinical risk prediction represents the next translational challenge. Given the inherent difficulties in obtaining direct human EUS biopsies, epidemiological tools and advanced analytical frameworks must be deployed to bridge this gap. Leveraging large-scale population health databases such as the National Health and Nutrition Examination Survey alongside machine learning approaches, including interpretable algorithms such as SHAP-based modeling, can help identify systemic metabolic phenotypes associated with increased susceptibility to SUI and may provide indirect evidence supporting the metabolic vulnerability framework. Furthermore, applying Mendelian Randomization frameworks to large genome-wide association study datasets is critical to establishing causal relationships between genetic proxies of systemic iron metabolism or lipid peroxidation and the clinical risk of progressive EUS degeneration.

Identifying biofluid-based biomarkers that can capture organ-specific metabolic damage non-invasively is essential for clinical translation. However, conventional systemic oxidative stress markers, such as serum MDA, lack sufficient pelvic-floor specificity to reflect localized EUS injury. Urinary or vaginal-fluid extracellular vesicles (EVs) may provide a minimally invasive source of candidate molecular signatures, but their attribution to the EUS remains technically challenging because of substantial source ambiguity, dilution by EVs derived from adjacent tissues, and the absence of validated EUS-specific surface or molecular markers. Future studies should therefore prioritize the identification of EUS-enriched cellular markers and tissue-specific EV signatures to enable selective EV enrichment and improve anatomical attribution. In parallel, high-resolution liquid chromatography–mass spectrometry could be used to characterize disease-associated oxidized phospholipid species and their diagnostic fragmentation patterns. These approaches may ultimately enable minimally invasive detection of early metabolic and regenerative dysfunction; however, urinary and vaginal EV signatures should currently be considered hypothesis-generating candidates rather than validated EUS-specific biomarkers.

Which cell populations within the EUS niche are most vulnerable to ferroptosis-associated stress determines where therapeutic intervention should be most precisely targeted. The distinct cellular constituents of the sphincter niche likely have vastly different stress response profiles. Cutting-edge technologies including single-cell RNA sequencing and spatial transcriptomics must be leveraged to construct cell type-specific expression atlases. This will clarify whether ferroptosis-associated damage initiates predominantly in mature myofibers, preferentially depletes the satellite cell pool, or primarily activates fibro-adipogenic progenitors.

What temporal trajectory metabolic stress and regenerative failure follow, and when the pathological process becomes irreversible, will define the optimal therapeutic window. The causal direction and temporal sequence between oxidative injury and ferroptotic execution remain unresolved. Dynamic tracking studies in multi-factor superimposed animal models—combining simulated parturition injury with metabolic syndrome or aging backgrounds—are needed. Incorporating temporal-specific genetic manipulation of iron–lipid–redox regulatory pathways within these models will disentangle cause from effect, determining optimal windows for resilience-restoring interventions.

## 10. Conclusions

In conclusion, the persistent degeneration paradox in SUI highlights that anatomical disruption is merely the initiating event, not the final determinant of disease trajectory. The ultimate fate of the external urethral sphincter is influenced by its systemic metabolic vulnerability signature.

We propose that iron–lipid–redox dysregulation serves as a critical molecular convergence point within this framework, linking mitochondrial dysfunction, lipid peroxidation, and impaired antioxidant defense to the failure of muscle maintenance. Rather than an isolated cell death pathway, this ferroptosis-associated metabolic stress synergizes with inflammatory, senescent, and ECM-remodeling networks may contribute to the transition from reversible functional impairment toward irreversible fibro-adipogenic degeneration.

This regenerative vulnerability model provides a new perspective for understanding why SUI often develops years after the initial pelvic insult and why anatomical restoration alone cannot fully prevent recurrence. Although direct demonstration of ferroptotic signaling in human EUS tissue remains an important future objective, converging evidence from skeletal muscle biology, aging research, and metabolic disorders supports systematic investigation of ferroptosis-related pathways as potential regulators of sphincter resilience.

Beyond mechanical reconstruction, future therapeutic strategies may require restoration of tissue metabolic fitness through modulation of mitochondrial quality control, redox homeostasis, lipid metabolism, and iron regulation. Ultimately, integrating biological vulnerability phenotyping with conventional anatomical assessment may enable a transition from a purely structural classification of SUI toward a precision framework based on regenerative capacity and disease susceptibility.

## Figures and Tables

**Table 1:** Clinical Phenotype–Metabolic Vulnerability Mapping in SUI.

Phenotype	Clinical Insult	Vulnerability Axis	Metabolic/Redox Checkpoint	Molecular Signature	EUS Relevance
Postpartum SUI	Parturition trauma: mechanical stretch, I/R, vascular occlusion, microhemorrhage (Section 4.1)	3.4 (mechanical/ischemic stress) + 3.3 (redox-sensitive membrane)	5.1 (iron homeostasis) + 5.3 (membrane vulnerability)	Acute Hb degradation → Fe^2+^; 15-LOX-1 ↑; GPX4 ↓; I/R-ROS burst; DAMP release	Direct: vaginal distension reduces EUS blood flow and causes I/R injury in rats [29,30]. 15-LOX-1/ACSL4 mechanisms extrapolated from cardiac I/R and skeletal muscle rhabdomyolysis [34,36] (X/M).
Aging-associated SUI	Aging: mitochondrial failure, iron dysregulation, chronic inflammation, hormonal transition (Section 4.2)	3.2 (limited regenerative reserve) + 3.1 (high energetic demand)	5.2 (redox buffering) + 5.1 (iron) + 5.4 (stem cell-stromal)	↑ Labile iron; ↓ GPX4; ↓ Nrf2-ARE; ↑ 4-HNE/MDA; mtDNA damage; PAX7+ senescence	Direct: age-related urethral oxidative stress and functional decline observed in rodent urethra [28,45] (D). Iron dysregulation, GPX4 decline, and satellite-cell senescence extrapolated from aged skeletal muscle [39,41,48] (M).
Obesity/Metabolic SUI	Systemic metabolic dysfunction: chronic inflammation, endothelial damage, oxidative stress, dyslipidemia (Section 4.3)	3.1 (high energetic demand) + 3.3 (redox-sensitive membrane)	5.3 (membrane vulnerability) + 5.2 (redox buffering)	↑ TNF-α/IL-6; ACSL4-mediated PUFA-PL enrichment; mitochondrial ROS overload; peroxidation-prone lipids	No direct EUS ferroptosis or lipidomic data. Metabolic syndrome linked to lower urinary tract mitochondrial damage in rats [56] (D for LUT dysfunction, H for ferroptosis). ACSL4-driven peroxidation and diabetic cardiomyopathy ferroptosis extrapolated from cardiac models [52,53] (X).
Neurogenic SUI	Neurogenic injury: pudendal nerve disruption, denervation metabolic reprogramming, EV propagation (Section 4.4)	3.2 (limited regenerative reserve) + 3.1 (high energetic demand)	5.2 (redox buffering) + 5.4 (stem cell-stromal)	↓ GPX4; impaired GSH utilization; mitochondrial decline; aberrant FAP activation; TGF-β-driven fibro-adipogenic transition	Direct: pudendal nerve dependence of EUS function established [57] (D). Denervation-induced mitochondrial decline and GPX4 impairment extrapolated from denervated skeletal muscle [58,65] (M). FAP activation in disused muscle [61] (M).

Evidence level key: D = Direct evidence from EUS/SUI models (human EUS tissue, primary EUS myoblasts, or validated animal EUS injury); M = Muscle-associated evidence from other striated muscle systems; X = Cross-tissue evidence from non-muscle organs; H = Conceptual inference from general biochemical or epidemiological principles. ↑ indicates an increase or upregulation, whereas ↓ indicates a decrease or downregulation in the corresponding molecular or biological feature. The ferroptosis-related molecular signatures in postpartum, aging, and neurogenic phenotypes currently rely primarily on M/X extrapolation; direct EUS-specific ferroptosis profiling is not yet available.

**Table 3:** Translational Strategies for Restoring Metabolic Resilience in EUS Degeneration.

Strategy	Rationale	Representative Approaches	Target Phenotype(s)	Evidence
Tier 1: Restoring metabolic resilience	Enhance endogenous adaptive capacity; activate Nrf2 antioxidant defense; optimize metabolic milieu	SFN (Nrf2-ARE activation, improves urethral function, elevates SOD/GSH-Px/CAT) [80]; 3HB (enhances autophagic/organelle remodeling in human EUS myoblasts) [110]	Aging-associated; Obesity/metabolic	Direct: rat SUI model (SFN); human EUS myoblast (3HB)
Tier 2: Preventing ferroptotic amplification	Inhibit ferroptotic cascade in tissues under oxidative stress; preserve functional reserve	DFO (reduces labile iron pool, attenuates degeneration) [75]; Fer-1 (reduces lipid peroxidation in mechanical stretch-induced pelvic floor damage) [34]	Postpartum; Neurogenic	Extrapolated: skeletal muscle atrophy (DFO); Direct: C2C12 + mouse pelvic floor muscle (Fer-1)
Tier 3: Rebuilding regenerative niche	Cell therapy and engineered EVs rebuild contractile tissue; redirect repair away from fibrosis	VEGF-mesoangioblasts (promote EUS neuromuscular regeneration in simulated vaginal delivery) [4]; ADSC-EVs + PEG hydrogel (upregulate Nrf2/GPX4, alleviate lipid peroxidation, promote M2 polarization) [116]	All phenotypes (universal endpoint); less effective in aging-associated (receptor senescence)	Direct: rat simulated birth injury (both)

## Data Availability

No new data were created or analyzed in this study. Data sharing is not applicable to this article.

## Abbreviations

The following abbreviations are used in this manuscript:

3HB	3-Hydroxybutyrate
4-HNE	4-Hydroxynonenal
ACSL4	Acyl-CoA synthetase long-chain family member 4
ADSC	Adipose-derived stem cell
ARE	Antioxidant response element
CAT	Catalase
DAMPs	Damage-associated molecular patterns
DFO	Deferoxamine
E-C	Excitation-contraction
ECM	Extracellular matrix
ETC	Electron transport chain
EUS	External urethral sphincter
EVs	Extracellular vesicles
FAPs	Fibro-adipogenic progenitors
Fer-1	Ferrostatin-1
FPN	Ferroportin
FTH1	Ferritin heavy chain 1
GPX4	Glutathione peroxidase 4
GSH	Glutathione
GSH-Px	Glutathione peroxidase
GWAS	Genome-wide association study
Hb	Hemoglobin
HMOX1	Heme oxygenase 1
I/R	Ischemia–reperfusion
IL-6	Interleukin-6
LOX	Lipoxygenase
Mb	Myoglobin
MDA	Malondialdehyde
MitoQ	Mitochondria-targeted antioxidant (MitoQ-like compounds)
mtDNA	Mitochondrial DNA
MUFA	Monounsaturated fatty acids
MyoD	Myogenic differentiation 1
MyoG	Myogenin
NADPH	Nicotinamide adenine dinucleotide phosphate
NCOA4	Nuclear receptor coactivator 4
NHANES	National Health and Nutrition Examination Survey
Nrf2	Nuclear factor erythroid 2-related factor 2
OXPHOS	Oxidative phosphorylation
PAX7	Paired box 7
PEG	Polyethylene glycol
PUFA	Polyunsaturated fatty acids
ROS	Reactive oxygen species
SFN	Sulforaphane
SHAP	SHapley Additive exPlanations
SLC3A2	Solute carrier family 3 member 2
SLC7A11	Solute carrier family 7 member 11
SOD	Superoxide dismutase
SUI	Stress urinary incontinence
TGF-β	Transforming growth factor-beta
TFR1	Transferrin receptor 1
TNF-α	Tumor necrosis factor-alpha
VEGF	Vascular endothelial growth factor

## References

[1] Zhu J., Pang H., Wang P., Chen Y., Li H., Liu Q., Wang L., Jin H., Gong L., Xie J. (2024). Female urinary incontinence in China after 15 years’ efforts: Results from large-scale nationwide surveys. Sci. Bull..

[2] Haylen B.T., de Ridder D., Freeman R.M., Swift S.E., Berghmans B., Lee J., Monga A., Petri E., Rizk D.E., Sand P.K. (2010). An International Urogynecological Association (IUGA)/International Continence Society (ICS) joint report on the terminology for female pelvic floor dysfunction. Neurourol. Urodyn..

[3] Patel U.J., Godecker A.L., Giles D.L., Brown H.W. (2022). Updated Prevalence of Urinary Incontinence in Women: 2015–2018 National Population-Based Survey Data. Female Pelvic Med. Reconstr. Surg..

[4] Mori da Cunha M., van der Veer B.K., Giacomazzi G., Mackova K., Cattani L., Koh K.P., Vande Velde G., Gijsbers R., Albersen M., Sampaolesi M. (2023). VEGF overexpressed mesoangioblasts enhance urethral and vaginal recovery following simulated vaginal birth in rats. Sci. Rep..

[5] Sajadi K.P., Lin D.L., Steward J.E., Balog B., Dissaranan C., Zaszczurynski P., Gill B.C., Jiang H.H., Kerns J.M., Damaser M.S. (2012). Pudendal nerve stretch reduces external urethral sphincter activity in rats. J. Urol..

[6] Wu A.K., Zhang X., Wang J., Ning H., Zaid U., Villalta J.D., Wang G., Banie L., Lin G., Lue T.F. (2018). Treatment of stress urinary incontinence with low-intensity extracorporeal shock wave therapy in a vaginal balloon dilation induced rat model. Transl. Androl. Urol..

[7] Carneiro A.S., Amorim S.G., Morais A.R., de Melo Correia S., Oliveira P., Silva V. (2026). Long-term course and predictors of postpartum urinary incontinence: A three-year cohort in Portuguese women. World J. Urol..

[8] Mitsui R., Miwa-Nishimura K., Hashitani H. (2025). Role of capillary rarefaction in age-related changes of external urethral sphincter muscle of female mice. Cell Tissue Res..

[9] Gosling J.A., Dixon J.S., Critchley H.O., Thompson S.A. (1981). A comparative study of the human external sphincter and periurethral levator ani muscles. Br. J. Urol..

[10] Tokunaka S., Fujii H., Hashimoto H., Yachiku S. (1993). Proportions of fiber types in the external urethral sphincter of young nulliparous and old multiparous rabbits. Urol. Res..

[11] Picard M., Hepple R.T., Burelle Y. (2012). Mitochondrial functional specialization in glycolytic and oxidative muscle fibers: Tailoring the organelle for optimal function. Am. J. Physiol. Cell Physiol..

[12] Kai H., Hata S., Hamamatsu N., Shinohara M., Matsuura K., Mimata H., Shin T. (2025). Metabolic dynamics of human external urethral sphincter myoblast differentiation and the effects of tricarboxylic acid cycle inhibition. Sci. Rep..

[13] Dixon S.J., Lemberg K.M., Lamprecht M.R., Skouta R., Zaitsev E.M., Gleason C.E., Patel D.N., Bauer A.J., Cantley A.M., Yang W.S. (2012). Ferroptosis: An iron-dependent form of nonapoptotic cell death. Cell.

[14] Stockwell B.R., Friedmann Angeli J.P., Bayir H., Bush A.I., Conrad M., Dixon S.J., Fulda S., Gascón S., Hatzios S.K., Kagan V.E. (2017). Ferroptosis: A Regulated Cell Death Nexus Linking Metabolism, Redox Biology, and Disease. Cell.

[15] Jiang X., Stockwell B.R., Conrad M. (2021). Ferroptosis: Mechanisms, biology and role in disease. Nat. Rev. Mol. Cell Biol..

[16] Doll S., Proneth B., Tyurina Y.Y., Panzilius E., Kobayashi S., Ingold I., Irmler M., Beckers J., Aichler M., Walch A. (2017). ACSL4 dictates ferroptosis sensitivity by shaping cellular lipid composition. Nat. Chem. Biol..

[17] Kagan V.E., Mao G., Qu F., Angeli J.P., Doll S., Croix C.S., Dar H.H., Liu B., Tyurin V.A., Ritov V.B. (2017). Oxidized arachidonic and adrenic PEs navigate cells to ferroptosis. Nat. Chem. Biol..

[18] Chen F., Kang R., Tang D., Liu J. (2024). Ferroptosis: Principles and significance in health and disease. J. Hematol. Oncol..

[19] Nakanishi T., Wakamatsu J., Nemoto S., Yamamoto A., Erickson L., Kawahara S. (2025). Differences in expression of ferroptosis-related genes and oxidative stress level between chicken breast and thigh muscles. Comp. Biochem. Physiol. Part A Mol. Integr. Physiol..

[20] Ji F., Lee H., Rheem H., Liu J., Kim J.H. (2025). Differential ferroptosis regulation in red and white gastrocnemius under obesity and its Attenuation by exercise and dietary restriction. Sci. Rep..

[21] Kuznetsov A.V., Margreiter R., Hagenbuchner J., Ausserlechner M.J. (2025). Energy metabolism in different skeletal muscles and muscle fibers: Implications for injury and dietary supplementation. Pflügers Arch. Eur. J. Physiol..

[22] Yang W.S., Kim K.J., Gaschler M.M., Patel M., Shchepinov M.S., Stockwell B.R. (2016). Peroxidation of polyunsaturated fatty acids by lipoxygenases drives ferroptosis. Proc. Natl. Acad. Sci. USA.

[23] Tsalouhidou S., Argyrou C., Theofilidis G., Karaoglanidis D., Orfanidou E., Nikolaidis M.G., Petridou A., Mougios V. (2006). Mitochondrial phospholipids of rat skeletal muscle are less polyunsaturated than whole tissue phospholipids: Implications for protection against oxidative stress. J. Anim. Sci..

[24] Cortie C.H., Hulbert A.J., Hancock S.E., Mitchell T.W., McAndrew D., Else P.L. (2015). Of mice, pigs and humans: An analysis of mitochondrial phospholipids from mammals with very different maximal lifespans. Exp. Gerontol..

[25] Lu X., Sharko O.L., Shmanai V.V., Shchepinov M.S., Markworth J.F. (2026). Deuterated polyunsaturated fatty acids alleviate in vitro skeletal muscle dysfunction induced by oxidative stress. Free Radic. Biol. Med..

[26] Mataix J., Quiles J.L., Huertas J.R., Battino M., Mañas M. (1998). Tissue specific interactions of exercise, dietary fatty acids, and vitamin E in lipid peroxidation. Free Radic. Biol. Med..

[27] Silva-Palacios A., Ostolga-Chavarría M., Zazueta C., Königsberg M. (2018). Nrf2: Molecular and epigenetic regulation during aging. Ageing Res. Rev..

[28] Alexandre E.C., Curiel A.A., Dias J.M., da Silva F.H., Antunes E., de Oliveira M.G. (2025). Aging impairs urethral function in male rats: Increased outlet resistance and oxidative stress. Auton. Neurosci. Basic Clin..

[29] Phull H.S., Pan H.Q., Butler R.S., Hansel D.E., Damaser M.S. (2011). Vulnerability of continence structures to injury by simulated childbirth. Am. J. Physiol. Ren. Physiol..

[30] Damaser M.S., Whitbeck C., Chichester P., Levin R.M. (2005). Effect of vaginal distension on blood flow and hypoxia of urogenital organs of the female rat. J. Appl. Physiol..

[31] Fang X., Cai Z., Wang H., Han D., Cheng Q., Zhang P., Gao F., Yu Y., Song Z., Wu Q. (2020). Loss of Cardiac Ferritin H Facilitates Cardiomyopathy via Slc7a11-Mediated Ferroptosis. Circ. Res..

[32] Dunn L.L., Kong S.M.Y., Tumanov S., Chen W., Cantley J., Ayer A., Maghzal G.J., Midwinter R.G., Chan K.H., Ng M.K.C. (2021). Hmox1 (Heme Oxygenase-1) Protects Against Ischemia-Mediated Injury via Stabilization of HIF-1α (Hypoxia-Inducible Factor-1α). Arterioscler. Thromb. Vasc. Biol..

[33] Ren X., Hu Y., Liu D., Wang G., Zhai P., Li Z., Sun L., Chen X., Lu M. (2026). Low-intensity pulsed ultrasound mitigates acute extremity compartment syndrome by mediating ferroptosis suppression. iScience.

[34] He Y., Huang G., Hong S., Zuo X., Zhao Z., Hong L. (2023). Ferrostatin-1 alleviates the damage of C2C12 myoblast and mouse pelvic floor muscle induced by mechanical trauma. Cell Death Discov..

[35] Başer Seçer M., Çïçek Güvendïk S., Çeliker Tosun O., Yavuz O., Kurt S., Tosun G., Basol Goksuluk M. (2025). The hidden power in the miracle of pregnancy: The effect of pelvic floor muscle training on fetal and fetal-maternal blood circulation and pelvic floor muscles during pregnancy, a randomized controlled trial. BMC Pregnancy Childbirth.

[36] Cai W., Liu L., Shi X., Liu Y., Wang J., Fang X., Chen Z., Ai D., Zhu Y., Zhang X. (2023). Alox15/15-HpETE Aggravates Myocardial Ischemia-Reperfusion Injury by Promoting Cardiomyocyte Ferroptosis. Circulation.

[37] He S., Li R., Peng Y., Wang Z., Huang J., Meng H., Min J., Wang F., Ma Q. (2022). ACSL4 contributes to ferroptosis-mediated rhabdomyolysis in exertional heat stroke. J. Cachexia Sarcopenia Muscle.

[38] Sommerfeld S.D., Cherry C., Schwab R.M., Chung L., Maestas D.R., Laffont P., Stein J.E., Tam A., Ganguly S., Housseau F. (2019). Interleukin-36γ-producing macrophages drive IL-17-mediated fibrosis. Sci. Immunol..

[39] Alves F.M., Ayton S., Bush A.I., Lynch G.S., Koopman R. (2023). Age-Related Changes in Skeletal Muscle Iron Homeostasis. J. Gerontol. Ser. A Biol. Sci. Med. Sci..

[40] Stanojevic N., Wohlgemuth S., Zeidan R.S., Lou X., Tamargo J.A., Bravo B., Newman J., Picca A., Marzetti E., Wu K. (2026). Iron dysregulation and mitochondrial dysfunction in aging: A longitudinal study on mobility decline in low- and high-functioning older adults. Exp. Gerontol..

[41] Zeidan R.S., Reinhard S., Picca A., Marzetti E., Leeuwenburgh C., Collins J.F., Anton S.D. (2026). Is Ferroptosis the Mechanistic Bridge Connecting Iron Dysregulation to Muscle Wasting and Functional Decline in Aging?. Aging Cell.

[42] Fang X., Ardehali H., Min J., Wang F. (2023). The molecular and metabolic landscape of iron and ferroptosis in cardiovascular disease. Nat. Rev. Cardiol..

[43] Chen X., Yu X., Zhong S., Sha P., Li R., Xu X., Liang N., Zhang L., Li L., Zhang J. (2026). ALDH2/eIF3E Interaction Modulates Protein Translation Critical for Cardiomyocyte Ferroptosis in Acute Myocardial Ischemia Injury. Circulation.

[44] Di Lorenzo R., Marzetti E., Coelho-Junior H.J., Calvani R., Pesce V., Landi F., Leeuwenburgh C., Picca A. (2025). Iron Metabolism and Muscle Aging: Where Ferritinophagy Meets Mitochondrial Quality Control. Cells.

[45] Otsubo A., Miyazato M., Oshiro T., Kimura R., Matsuo T., Miyata Y., Sakai H. (2021). Age-associated bladder and urethral coordination impairment and changes in urethral oxidative stress in rats. Life Sci..

[46] Huang Y., Wu B., Shen D., Chen J., Yu Z., Chen C. (2021). Ferroptosis in a sarcopenia model of senescence accelerated mouse prone 8 (SAMP8). Int. J. Biol. Sci..

[47] Johnson A.L., Bevington R.T., Parise G. (2026). Senescence as a regulatory mechanism in skeletal muscle repair in young mice. Am. J. Physiol. Cell Physiol..

[48] Sousa-Victor P., Gutarra S., Garcia-Prat L., Rodriguez-Ubreva J., Ortet L., Ruiz-Bonilla V., Jardi M., Ballestar E., Gonzalez S., Serrano A.L. (2014). Geriatric muscle stem cells switch reversible quiescence into senescence. Nature.

[49] Shang X., Fu Y., Jin X., Wang C., Wang P., Guo P., Wang Y., Yan S. (2023). Association of overweight, obesity and risk of urinary incontinence in middle-aged and older women: A meta epidemiology study. Front. Endocrinol..

[50] Huang H., Han X., Liu Q., Xue J., Yu Z., Miao S. (2022). Associations between metabolic syndrome and female stress urinary incontinence: A meta-analysis. Int. Urogynecol. J..

[51] Hadizadeh A., Chill H.H., Leffelman A., Burgos A., Chang C., Lee J., Paya-Ten C., Goldberg R.P., Abramowitch S.D., Rostaminia G. (2026). Impact of bariatric surgery on pelvic floor dysfunction symptoms: A systematic review and meta-analysis of prospective Studies. Surg. Obes. Relat. Dis. Off. J. Am. Soc. Bariatr. Surg..

[52] Xiang H., Lyu Q., Chen S., Ouyang J., Xiao D., Liu Q., Long H., Zheng X., Yang X., Lu H. (2024). PACS2/CPT1A/DHODH signaling promotes cardiomyocyte ferroptosis in diabetic cardiomyopathy. Cardiovasc. Diabetol..

[53] Zhao Y., Pan B., Lv X., Chen C., Li K., Wang Y., Liu J. (2023). Ferroptosis: Roles and molecular mechanisms in diabetic cardiomyopathy. Front. Endocrinol..

[54] Yang H., Xiao G., Wang D., Xiong T., Wang J., Jing X., Xiong B., Xie J., Liu B., She Q. (2025). Inhibition of HMOX1 alleviates diabetic cardiomyopathy by targeting ferroptosis. Acta Biochim. Biophys. Sin..

[55] Tian M., Huang X., Li M., Lou P., Ma H., Jiang X., Zhou Y., Liu Y. (2024). Ferroptosis in diabetic cardiomyopathy: From its mechanisms to therapeutic strategies. Front. Endocrinol..

[56] Chung S.D., Chien C.T., Yu H.J. (2013). Alterations in peripheral purinergic and muscarinic signaling of rat bladder after long-term fructose-induced metabolic syndrome. Eur. J. Nutr..

[57] Lee C.L., Lee J., Park J.M., Na H.S., Shin J.H., Na Y.G., Kim K.H. (2021). Sophisticated regulation of micturition: Review of basic neurourology. J. Exerc. Rehabil..

[58] Triolo M., Bhattacharya D., Hood D.A. (2022). Denervation induces mitochondrial decline and exacerbates lysosome dysfunction in middle-aged mice. Aging.

[59] Yang J., Balog B., Deng K., Hanzlicek B., Rietsch A., Kuang M., Hatakeyama S., Lach-Trifilieff E., Zhu H., Damaser M.S. (2020). Therapeutic potential of muscle growth promoters in a stress urinary incontinence model. Am. J. Physiol. Ren. Physiol..

[60] Liu X., Chen Y., Zhang L., Qi Z., Yang L., Huang C., Wang L., Lin D. (2025). Taurine Attenuates Disuse Muscle Atrophy Through Modulation of the xCT-GSH-GPX4 and AMPK-ACC-ACSL4 Pathways. Antioxidants.

[61] Tan J., Li Y., Zhang J., Qi B., Chen J., Sun Y. (2025). Role of aberrant activated fibro/adipogenic progenitors and suppressed ferroptosis in disused skeletal muscle atrophy and fatty infiltration. J. Mol. Med..

[62] Tao L., Wang J., Wang K., Liu Q., Li H., Xu S., Gu C., Zhu Y. (2024). Exerkine FNDC5/irisin-enriched exosomes promote proliferation and inhibit ferroptosis of osteoblasts through interaction with Caveolin-1. Aging Cell.

[63] Huang M., Cheng S., Li Z., Chen J., Wang C., Li J., Zheng H. (2024). Preconditioning Exercise Inhibits Neuron Ferroptosis and Ameliorates Brain Ischemia Damage by Skeletal Muscle-Derived Exosomes via Regulating miR-484/ACSL4 Axis. Antioxid. Redox Signal..

[64] Liu Z., He Y., Hao C. (2026). Exosomal MiR-223-3p from Adipose-Derived Stem Cells Alleviates Hypoxia/Reoxygenation-Induced Ferroptosis of H9c2 Cells by Inhibiting TFRC. Int. Heart J..

[65] Liu Y., Xu C., Wang Z., Duan K., Chen L., Yu H., Guan J. (2025). Microvascular Endothelial Cell-Derived Exosomes Decrease Osteoclastogenesis by Restraining Osteoclast Ferroptosis through the USP13/NRF2/GPX4 Pathway. Crit. Rev. Immunol..

[66] Yuan Y., Mei Z., Qu Z., Li G., Yu S., Liu Y., Liu K., Shen Z., Pu J., Wang Y. (2023). Exosomes secreted from cardiomyocytes suppress the sensitivity of tumor ferroptosis in ischemic heart failure. Signal Transduct. Target. Ther..

[67] Yang W.S., SriRamaratnam R., Welsch M.E., Shimada K., Skouta R., Viswanathan V.S., Cheah J.H., Clemons P.A., Shamji A.F., Clish C.B. (2014). Regulation of ferroptotic cancer cell death by GPX4. Cell.

[68] Stockwell B.R. (2022). Ferroptosis turns 10: Emerging mechanisms, physiological functions, and therapeutic applications. Cell.

[69] Zhang G., Zhang Y., Shen Y., Wang Y., Zhao M., Sun L. (2021). The Potential Role of Ferroptosis in Alzheimer’s Disease. J. Alzheimer’s Dis. JAD.

[70] Ding H., Chen S., Pan X., Dai X., Pan G., Li Z., Mai X., Tian Y., Zhang S., Liu B. (2021). Transferrin receptor 1 ablation in satellite cells impedes skeletal muscle regeneration through activation of ferroptosis. J. Cachexia Sarcopenia Muscle.

[71] Chen Y., Guo Y., Zhao Z., Yang S., Guo B., Xu J. (2026). Current Progress in the Role of Ferroptosis in Skeletal Muscle Atrophy. Mediat. Inflamm..

[72] Ru Q., Li Y., Zhang X., Chen L., Wu Y., Min J., Wang F. (2025). Iron homeostasis and ferroptosis in muscle diseases and disorders: Mechanisms and therapeutic prospects. Bone Res..

[73] Santana-Codina N., Gikandi A., Mancias J.D. (2021). The Role of NCOA4-Mediated Ferritinophagy in Ferroptosis. Adv. Exp. Med. Biol..

[74] Peng T., Zhang Z., Wei J., Ding N., Liang W., Tang X. (2026). Systemic Integrative Mechanisms and Intervention Strategies in Exercise-Induced Skeletal Muscle Damage: Evidence from Animal, Clinical, and Multi-Omics Studies. Int. J. Mol. Sci..

[75] Jeong J.Y., Son Y., Kim Y., Lee N., Kim Y., Heo Y.J., Choi S.E., Choi J., Han S.J., Jeon J. (2025). Deferoxamine prevents dexamethasone-induced muscle atrophy by reducing MuRF1 and atrogin-1. Front. Pharmacol..

[76] Fantini G.A., Yoshioka T. (1993). Deferoxamine prevents lipid peroxidation and attenuates reoxygenation injury in postischemic skeletal muscle. Am. J. Physiol..

[77] Sun C.C., Xiao J.L., Sun C., Tang C.F. (2024). Ferroptosis and Its Potential Role in the Physiopathology of Skeletal Muscle Atrophy. Int. J. Mol. Sci..

[78] Wang L., Liu Y., Du T., Yang H., Lei L., Guo M., Ding H.F., Zhang J., Wang H., Chen X. (2020). ATF3 promotes erastin-induced ferroptosis by suppressing system Xc^−^. Cell Death Differ..

[79] Khan S.U., Halawi M.H., Almehmadi M., Ibrahim E.H., Taha R., Alsyaad K.M., Ahmed A.E., Al-Doaiss A.A., Thornbury W. (2026). NRF2-mediated anti-ferroptotic pathways in diabetic cardiomyopathy: Mechanistic insights, therapeutic advances, and challenges in cardiovascular protection. Curr. Probl. Cardiol..

[80] Wan X., Liu C., Chen Y.B., Gu M., Cai Z.K., Chen Q., Wang Z. (2017). Sulforaphane Treatment of Stress Urinary Incontinence Via the Nrf2-ARE Pathway in a Rat Model. Cell. Physiol. Biochem. Int. J. Exp. Cell. Physiol. Biochem. Pharmacol..

[81] Zheng H., Liang Y., Tian Y. (2026). Ferroptosis-driven immune remodeling in heart failure: The central role of macrophage reprogramming and impaired efferocytosis. J. Nanobiotechnol..

[82] Chen X., Xiong Z., Xie Y., Ruan K., Huang G., Mao J. (2026). Crosstalk between ion homeostasis and lipid peroxidation: Mechanisms and translational opportunities in regulated cell death. Pharmacol. Res..

[83] Łoboda A., Dulak J. (2023). Nuclear Factor Erythroid 2-Related Factor 2 and Its Targets in Skeletal Muscle Repair and Regeneration. Antioxid. Redox Signal..

[84] Rajasekaran N.S., Shelar S.B., Jones D.P., Hoidal J.R. (2020). Reductive stress impairs myogenic differentiation. Redox Biol..

[85] Gu X., Fan L., Ke R., Chen Y. (2018). rHGF interacts with rIGF-1 to activate the satellite cells in the striated urethral sphincter in rats: A promising treatment for urinary incontinence?. Arch. Gynecol. Obstet..

[86] Tran L., Xie B., Assaf E., Ferrari R., Pipinos I.I., Casale G.P., Mota Alvidrez R.I., Watkins S., Sachdev U. (2023). Transcriptomic Profiling Identifies Ferroptosis-Related Gene Signatures in Ischemic Muscle Satellite Cells Affected by Peripheral Artery Disease-Brief Report. Arterioscler. Thromb. Vasc. Biol..

[87] Che Y., Li J., Wang P., Yu W., Lin J., Su Z., Ye F., Zhang Z., Xu P., Xie Z. (2023). Iron deficiency-induced ferritinophagy impairs skeletal muscle regeneration through RNF20-mediated H2Bub1 modification. Sci. Adv..

[88] Wu R., Huang C., Wu Q., Jia X., Liu M., Xue Z., Qiu Y., Niu X., Wang Y. (2019). Exosomes secreted by urine-derived stem cells improve stress urinary incontinence by promoting repair of pubococcygeus muscle injury in rats. Stem Cell Res. Ther..

[89] Yang B., Li M., Lei H., Xu Y., Li H., Gao Z., Guan R., Xin Z. (2019). Low Intensity Pulsed Ultrasound Influences the Myogenic Differentiation of Muscle Satellite Cells in a Stress Urinary Incontinence Rat Model. Urology.

[90] Molloy A.M., Mills J.L., McPartlin J., Kirke P.N., Scott J.M., Daly S. (2002). Maternal and fetal plasma homocysteine concentrations at birth: The influence of folate, vitamin B12, and the 5,10-methylenetetrahydrofolate reductase 677C→T variant. Am. J. Obstet. Gynecol..

[91] Streltsova O., Kuyarov A., Molvi M., Zubova S., Lazukin V., Tararova E., Kiseleva E. (2020). New Approaches in the Study of the Pathogenesis of Urethral Pain Syndrome. Diagnostics.

[92] Fang W., Du X., Yang R., Liu M., Yang M., Jin Y., Gao G., Fu Q., Wang Y. (2025). Injectable therapeutic system incorporating neurogenesis-programmed stem cells concomitantly promoting muscle regeneration treats stress urinary incontinence. Nat. Commun..

[93] Lemos D.R., Babaeijandaghi F., Low M., Chang C.K., Lee S.T., Fiore D., Zhang R.H., Natarajan A., Nedospasov S.A., Rossi F.M. (2015). Nilotinib reduces muscle fibrosis in chronic muscle injury by promoting TNF-mediated apoptosis of fibro/adipogenic progenitors. Nat. Med..

[94] Yin K., Zhang C., Deng Z., Wei X., Xiang T., Yang C., Chen C., Chen Y., Luo F. (2024). FAPs orchestrate homeostasis of muscle physiology and pathophysiology. FASEB J. Off. Publ. Fed. Am. Soc. Exp. Biol..

[95] Su H., Cantrell A.C., Chen J.X., Gu W., Zeng H. (2023). SIRT3 Deficiency Enhances Ferroptosis and Promotes Cardiac Fibrosis via p53 Acetylation. Cells.

[96] Zhang X., Habiballa L., Aversa Z., Ng Y.E., Sakamoto A.E., Englund D.A., Pearsall V.M., White T.A., Robinson M.M., Rivas D.A. (2022). Characterization of cellular senescence in aging skeletal muscle. Nat. Aging.

[97] Moiseeva V., Cisneros A., Sica V., Deryagin O., Lai Y., Jung S., Andrés E., An J., Segalés J., Ortet L. (2023). Senescence atlas reveals an aged-like inflamed niche that blunts muscle regeneration. Nature.

[98] Xie X., Zhao J., Wu T., Yang Y., Zong Y., Li L., Gao Y., Jiang L., Cao Y., Shen L. (2025). Aging-Associated CD55^+^ Fibroblasts Promote Chronic Inflammation and ECM Dysregulation in Sarcopenia. J. Inflamm. Res..

[99] Bonanni R., Falvino A., Matticari A., Rinaldi A.M., D’Arcangelo G., Cifelli P., Iundusi R., Gasbarra E., Tancredi V., Cariati I. (2025). Targeting ERRs to counteract age-related muscle atrophy associated with physical inactivity: A pilot study. Front. Physiol..

[100] Jimenez-Gutierrez G.E., Martínez-Gómez L.E., Martínez-Armenta C., Pineda C., Martínez-Nava G.A., Lopez-Reyes A. (2022). Molecular Mechanisms of Inflammation in Sarcopenia: Diagnosis and Therapeutic Update. Cells.

[101] Li Y., Li C., Zhou Q., Liu X., Qiao Y., Xie T., Sun H., Ong M.T., Wang H. (2025). Multiomics and cellular senescence profiling of aging human skeletal muscle uncovers Maraviroc as a senotherapeutic approach for sarcopenia. Nat. Commun..

[102] Meng F., He J., Zhang X., Lyu W., Wei R., Wang S., Du Z., Wang H., Bi J., Hua X. (2025). Histone Lactylation Antagonizes Senescence and Skeletal Muscle Aging by Modulating Aging-Related Pathways. Adv. Sci..

[103] Piccoli M., Mornatti L., Lavota I., Risuglia M., Creo P., Vizzino E., Rota P., Tarantino A., Mangiavini L., Ciconte G. (2026). The HIF-1α Pathway Regulates Satellite Cell Fate During Aging Through Histone Lactylation. Aging Cell.

[104] Gustafsson T., Ulfhake B. (2024). Aging Skeletal Muscles: What Are the Mechanisms of Age-Related Loss of Strength and Muscle Mass, and Can We Impede Its Development and Progression?. Int. J. Mol. Sci..

[105] Bourrant P.E., Yee E.M., Fennel Z.J., Castro R.J., Skiles C.M., Petrocelli J.J., de Hart N., Lesniewski L.A., Beaudin A.E., Funai K. (2026). Multicellular senescence impairs skeletal muscle recovery following disuse in aging. Sci. Adv..

[106] Sacco E., Campetella M., Marino F., Gavi F., Moretto S., Pastorino R., Bizzarri F.P., Pierconti F., Gandi C., Bientinesi R. (2025). Preoperative Risk Factors for Failure After Fixed Sling Implantation for Postprostatectomy Stress Urinary Incontinence (FORESEE): A Systematic Review and Meta-analysis. Eur. Urol. Open Sci..

[107] Terrell K., Choi S., Choi S. (2023). Calcium’s Role and Signaling in Aging Muscle, Cellular Senescence, and Mineral Interactions. Int. J. Mol. Sci..

[108] Liu C., Wong P.Y., Wang Q., Wong H.Y., Huang T., Cui C., Zhang N., Cheung W.H., Wong R.M.Y. (2024). Short-chain fatty acids enhance muscle mass and function through the activation of mTOR signalling pathways in sarcopenic mice. J. Cachexia Sarcopenia Muscle.

[109] Kanaan M.N., Pileggi C.A., Karam C.Y., Kennedy L.S., Fong-McMaster C., Cuperlovic-Culf M., Harper M.E. (2024). Cystine/glutamate antiporter xCT controls skeletal muscle glutathione redox, bioenergetics and differentiation. Redox Biol..

[110] Kai H., Hata S., Hamamatsu N., Shiraishi R., Shin T. (2026). Physiological medium and 3-hydroxybutyrate modulate autophagy-linked organelle remodeling in human external urethral sphincter myoblasts. Sci. Rep..

[111] Pfeuti C.K., Przydacz M., Linder B.J. (2026). Urodynamics for Female Stress Urinary Incontinence: When and Why. Neurourol. Urodyn..

[112] Moris L., Heesakkers J., Nitti V., O’Connell H.E., Peyronnet B., Serati M., Omar M.I., Harding C. (2025). Prevalence, Diagnosis, and Management of Stress Urinary Incontinence in Women: A Collaborative Review. Eur. Urol..

[113] Tratenšek A., Locatelli I., Grabnar I., Drobne D., Vovk T. (2024). Oxidative stress-related biomarkers as promising indicators of inflammatory bowel disease activity: A systematic review and meta-analysis. Redox Biol..

[114] Zhou H., Zhu C., Li Y., Zhao F., Feng Q., Liu S., Jia S., Ji J., Ye L., Zhai G. (2025). Exosome/liposome hybrid nanovesicles for enhanced phototherapy and boosted anti-tumor immunity against melanoma. Eur. J. Med. Chem..

[115] Cho S., Tam E., Nguyen K., Lei Y., Fillebeen C., Pantopoulos K., Sung H.K., Sweeney G. (2025). ω-6 PUFA-enriched membrane phospholipid composition of cardiomyocytes increases the susceptibility to iron-induced ferroptosis and inflammation. Apoptosis Int. J. Program. Cell Death.

[116] Wang Q., Li Y., Zhang Y., Wu X., Wang S., Sun X., Wang J. (2025). Extracellular Vesicles from Adipose-Derived Mesenchymal Stem Cells Combined with PEG Hydrogel Alleviate Maternal Simulated Birth Injury in a Rat Model. Adv. Healthc. Mater..

[117] Andrysiak K., Machaj G., Priesmann D., Woźnicka O., Martyniak A., Ylla G., Krüger M., Pyza E., Potulska-Chromik A., Kostera-Pruszczyk A. (2024). Dysregulated iron homeostasis in dystrophin-deficient cardiomyocytes: Correction by gene editing and pharmacological treatment. Cardiovasc. Res..

